# Tenascin-C drives persistence of organ fibrosis

**DOI:** 10.1038/ncomms11703

**Published:** 2016-06-03

**Authors:** Swati Bhattacharyya, Wenxia Wang, Luisa Morales-Nebreda, Gang Feng, Minghua Wu, Xiaodong Zhou, Robert Lafyatis, Jungwha Lee, Monique Hinchcliff, Carol Feghali-Bostwick, Katja Lakota, G. R. Scott Budinger, Kirtee Raparia, Zenshiro Tamaki, John Varga

**Affiliations:** 1 Northwestern University Feinberg School of Medicine, Chicago, Illinois 60611, USA; 2 University of Texas Medical School at Houston, Houston, Texas 77030, USA; 3 Boston University School of Medicine, Boston, Massachusetts 02215, USA; 4 Medical University of South Carolina, Charleston, South Carolina 29225, USA

## Abstract

The factors responsible for maintaining persistent organ fibrosis in systemic sclerosis (SSc) are not known but emerging evidence implicates toll-like receptors (TLRs) in the pathogenesis of SSc. Here we show the expression, mechanism of action and pathogenic role of endogenous TLR activators in skin from patients with SSc, skin fibroblasts, and in mouse models of organ fibrosis. Levels of tenascin-C are elevated in SSc skin biopsy samples, and serum and SSc fibroblasts, and in fibrotic skin tissues from mice. Exogenous tenascin-C stimulates collagen gene expression and myofibroblast transformation via TLR4 signalling. Mice lacking tenascin-C show attenuation of skin and lung fibrosis, and accelerated fibrosis resolution. These results identify tenascin-C as an endogenous danger signal that is upregulated in SSc and drives TLR4-dependent fibroblast activation, and by its persistence impedes fibrosis resolution. Disrupting this fibrosis amplification loop might be a viable strategy for the treatment of SSc.

## Abstract (web summary)

Systemic sclerosis (SSc) is a fibrotic disease affecting multiple organs. Here the authors use patient samples plus mouse studies to show a central role for tenascin C as a TLR4 activator responsible for persistence of fibrosis in the context of SSc and SSc-like disease.

## Graphical abstract

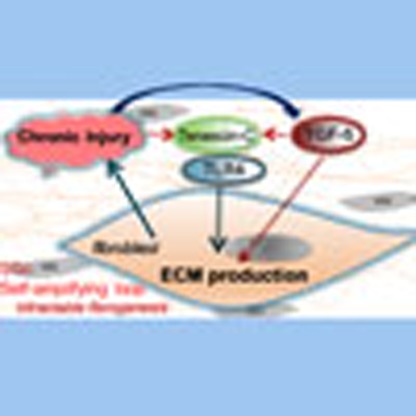

Fibrosis underlies the morbidity and mortality in systemic sclerosis (SSc) and many other human diseases, and currently has no effective therapy^1^. Although fibrosis is a complex, multicellular dynamic process, ultimately it is dysregulated wound healing characterized by the failure of lesional fibroblasts to enter quiescence. Transforming growth factor-β (TGF- β), along with cytokines such as interleukin IL-6 and growth factors including Wnt ligands and platelet-derived growth factor, is implicated as a key factor initiating pathological tissue remodelling in SSc. However, the mechanism responsible for the persistence of fibrotic response in SSc, and the factors maintaining activation of lesional tissue fibroblasts, is not well understood^2^. Recent transcriptome analyses of SSc skin biopsies reveal that progressive skin involvement is accompanied by aberrant expression of genes involved in immunity and tissue remodelling^3^,^4^. Markers of innate immunity are prominent in patients with SSc with both early- and late-stage disease, pointing to a potential role of toll-like receptors (TLRs) and TLR-mediated responses in driving pathogenesis. Toll-like receptors expressed on both macrophages and stromal cells can be activated by endogenous ligands called damage-associated molecular patterns (DAMPs) generated during tissue injury. Endogenous TLR ligands comprise a large and diverse family of molecules acting as danger signals to alert the host to the presence of tissue damage. These DAMPs trigger TLR-mediated inflammatory and fibrotic responses that are beneficial when properly regulated, but harmful when chronic and de-regulated^5^.

Our studies seeking to understand the persistence of fibrosis in SSc implicate tenascin-C as an endogenous TLR4 ligand with potent profibrotic activity and a potential pathogenic role in SSc. Tenascin-C is a multifunctional hexameric extracellular matrix (ECM) glycoprotein that undergoes extensive alternate splicing to generate multiple isoforms^6^. Normally tenascin-C is under tight spatial and temporal regulation, with prominent expression during embryogenesis but undetectable in most healthy adult tissues, and transient re-expression during wound healing and dynamic tissue remodelling. By contrast, persistent tenascin-C accumulation occurs in a variety of chronic pathological conditions^7^. The present results indicate that tenascin-C is persistently elevated in both the affected tissue and circulation in SSc patients, and is capable of inducing potent fibrotic responses mediated via TLR4. Moreover, genetic deletion of tenascin-C in mice is associated with attenuated cutaneous and lung inflammation and fibrosis, and accelerated fibrosis resolution. These results implicate tenascin-C as an important mediator of persistent tissue fibrosis in SSc, and suggest that blocking TLR4-dependent fibroblast activation might represent a novel strategy for therapeutic intervention.

## Results

### Tenascin-C levels are elevated in patients with SSc

**Figure 1:**
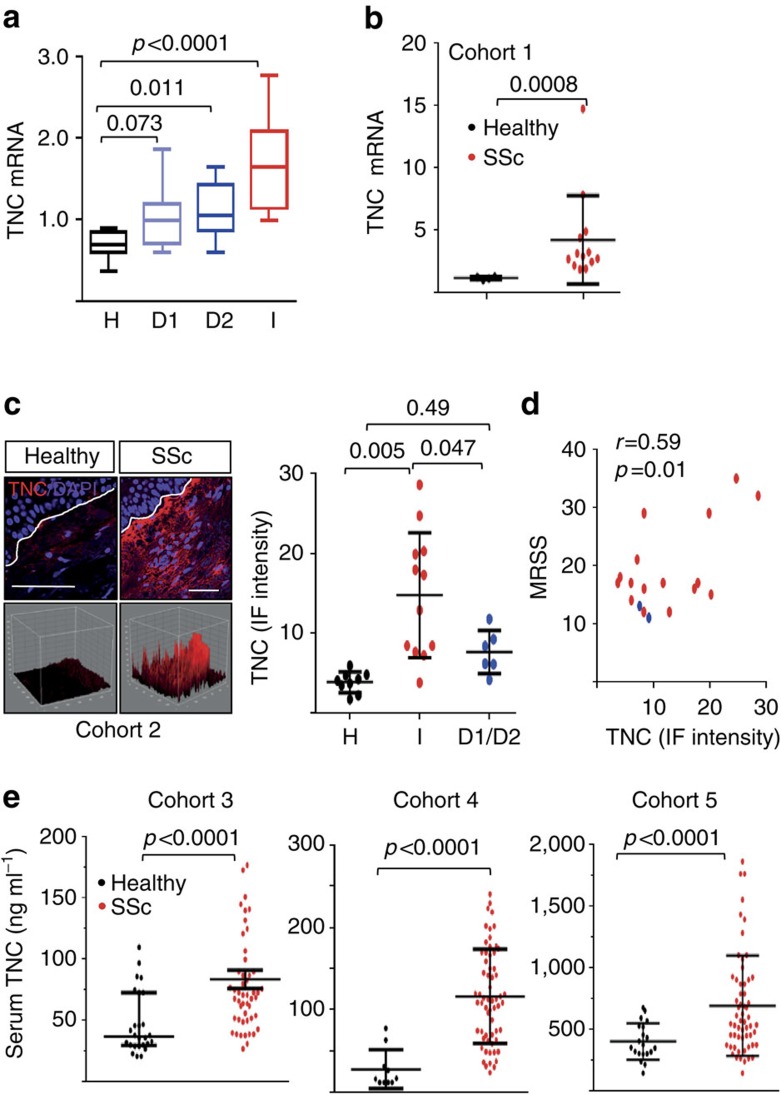
Tenascin-C is elevated in SSc. (**a**) Tenascin-C (TNC) mRNA expression in SSc biopsy-derived microarray data sets (GSE32413); levels in individual intrinsic gene subsets (H, healthy controls; D1, diffuse 1; D2, diffuse 2; I, inflammatory) are shown as box plots spanning values from 25–75 percentile; horizontal lines represent median, maximum and minimum values. One-way analysis of variance (ANOVA) followed by Sidak's multiple comparison test. (**b**) RNA from SSc (*n*=14) and healthy adult (*n*=4) skin biopsies examined by real-time qPCR. Results, expressed relative to GAPDH, are means±s.d. of duplicate determinations. *P<0.05, Mann–Whitney *U*-test. (**c**) Immunofluorescence microscopy. SSc (*n*=18) and healthy control (*n*=9) skin biopsies were stained with antibodies to tenascin-C. Left panels, representative confocal immunofluorescence images; dotted lines indicate dermal–epidermal junction. Scale bar, 50 μm. Bottom, 3D plots. Right panel, quantitation of fluorescence intensity. Each point represents mean immunofluorescence intensity from four randomly selected h.p.f.'s in skin biopsies from healthy controls, and SSc biopsies classified as inflammatory (I), or diffuse-proliferative (D1/D2). Results, expressed, are means±s.d. of quadruplet determinations from a single subject. One-way ANOVA followed by Sidak's multiple comparison test. (**d**) Tissue levels of tenascin-C correlate with MRSS. Each dot is a single subject (red, early-stage SSc; blue, late-stage SSc). Pearson's correlation. (**e**) Circulating tenascin-C in SSc. Serum levels in Cohort 3 (50 SSc patients and 26 healthy controls), Cohort 4 (62 SSc patients and 10 healthy controls) and Cohort 5 (38 SSc patients and 12 healthy controls) determined by enzyme-linked immunosorbent assay. Each dot is the mean±s.d. of triplicate determinations from a single subject. Mann–Whitney *U*-test.

Initial studies sought to identify damage-associated molecular patterns showing aberrant expression in SSc patients. For this purpose, we compared SSc and healthy control skin biopsies for the expression of six endogenous TLR ligands that had been implicated in sterile inflammation (Supplementary Table 1). These studies showed, for the first time, a marked increase in tenascin-C in lesional SSc skin biopsies, focusing our subsequent studies on this matricellular glycoprotein with both functional and structural roles. The expression of tenascin-C is normally under tight spatiotemporal regulation. To characterize its expression in SSc, we initially turned to a publicly available transcriptome data set (GSE32413) comprising skin biopsies from 13 SSc patients and 9 healthy controls^8^. Levels of tenascin-C mRNA were found to be significantly elevated in SSc skin biopsies mapping to the previously defined diffuse and inflammatory intrinsic gene expression subsets compared with skin biopsies from healthy controls (Fig. 1a). The inflammatory intrinsic gene expression subset is enriched in genes involved in macrophage function, innate immunity, TLR signalling and inflammation^3^. Biopsies clustering with the inflammatory intrinsic SSc subset, accounting for ∼50% of all SSc biopsies, showed the highest tenascin-C expression (*P*<0.0001 compared with healthy controls, Mann–Whitney *U*). Tenascin-C expression showed strong correlation (*r*=0.57, *P*=0.011) with TLR4, as well as IL-6 (*r*=0.40, *P*=0.0036; Pearson's correlation), a proinflammatory profibrotic cytokine that is a direct target of TLR4 in fibroblasts (Supplementary Fig. 1A). Elevated tenascin-C mRNA expression in these skin biopsies was accompanied by significant elevation of the fibrotic genes *COL1A1*, *COL1A2*, *COL1A6*, *PAI-1* and *COMP* (*P*<0.01 compared to healthy controls, Mann–Whitney *U*). A replication study with an independent cohort of 27 patients (including 3 with localized scleroderma or morphea) and 6 healthy controls (GSE9285) confirmed elevated tenascin-C expression in the inflammatory intrinsic subset biopsies (comprising 34% of SSc biopsies) compared with healthy control biopsies (*P*=0.030, Mann–Whitney *U*)^9^ (Supplementary Fig. 1B). Finally, a meta-analysis of three distinct transcriptome data sets (GSE56038 and GSE59785) comprising skin biopsies from a total of 80 SSc patients (70 diffuse cutaneous SSc (dcSSc) and 10 limited cutaneous (lcSSc)) and 26 healthy controls confirmed significantly elevated tenascin-C mRNA (*P*<0.00010, Mann–Whitney *U*) in the inflammatory gene expression subset biopsies (comprising 36% of all SSc biopsies) compared with healthy control biopsies. To further examine the expression of tenascin-C mRNA in SSc, we determined mRNA levels directly in SSc skin biopsies. By real-time quantitative PCR (qPCR) we show, for the first time, elevated levels of tenascin-C mRNA in SSc (*n*=13; Cohort 1, Table 1) compared with healthy control (*n*=4) skin biopsies (Fig. 1b). Notably, among skin biopsies classified as inflammatory (78% of the total in this group), tenascin-C levels showed strong correlation with the modified Rodnan Skin Score (MRSS; *r*=0.73, *P*=0.03, Spearman's rank correlation).

To evaluate tenascin-C protein accumulation in the skin, immunofluorescence microscopy was performed. Biopsies from healthy controls (*n*=9) showed barely detectable tenascin-C, in agreement with our preliminary results above, and confirming previous reports of largely absent tenascin-C in normal human skin^10^. In contrast, tenascin-C was readily detectable in SSc skin biopsies (*n*=18; Cohort 2, Table 1), with most prominent deposition in the papillary dermis subjacent to the basement membrane (Fig. 1c). Tenascin-C protein levels were highest in biopsies classified as inflammatory based on gene expression subsets, and showed moderate correlation with the MRSS (Fig. 1d, *r*=0.59, *P*=0.01; Pearson's correlation). In contrast, no correlation of tenascin-C with disease duration or change in MRSS over 6 months was found. Fibroblast lines established from four of six skin SSc biopsies showed increased tenascin-C expression by both qPCR, and by immunofluorescence, compared with healthy controls (Supplementary Fig. 1C).

Next, we sought to determine levels of circulating tenascin-C in SSc. In an initial analysis (Cohort 3, Table 2), serum tenascin-C levels were significantly elevated in SSc patients compared with healthy controls (mean, 71 ng ml^−1^, interquartile range 51.7–92.5 versus 37 ng ml^−1^, interquartile range 29–73; Fig. 1e). Moreover, 82% of dcSSc patients had elevated tenascin-C (>105.4 ng ml^−1^; defined as ≥95% confidence interval for healthy subjects, Table 3). Importantly, tenascin-C levels were significantly higher in patients with dcSSc compared with lcSSc, and showed correlation with MRSS (*r*=0.421, *P*=0.04). In contrast, there was no difference in disease duration, autoantibody profile or presence of interstitial lung disease, between subjects with normal versus elevated serum tenascin-C (Table 3). A validation study (Cohort 4, 62 SSc patients and age- and sex-matched 10 healthy controls) confirmed significantly elevated serum levels tenascin-C in SSc (Fig. 1e), with the highest levels in patients with SSc-interstitial lung disease (ILD) (ILD versus no ILD *P*=0.017), confirming results from a previous study (Supplementary Table 2) (ref. 11). An additional validation study confirmed elevated serum tenascin-C levels, even when adjusted for age, sex and race (*P*<0.0001) in an independent SSc cohort (Cohort 5; Table 4 and Supplementary Table 3). In cohorts 4 and 5, levels of serum tenascin-C did not show correlation with the MRSS. Together, these results indicate for the first time that tenascin-C gene expression and levels are elevated in the skin, in explanted fibroblasts, and in the circulation, in patients with SSc.

### Tenascin-C expression is enhanced by profibrotic cytokines

**Figure 2:**
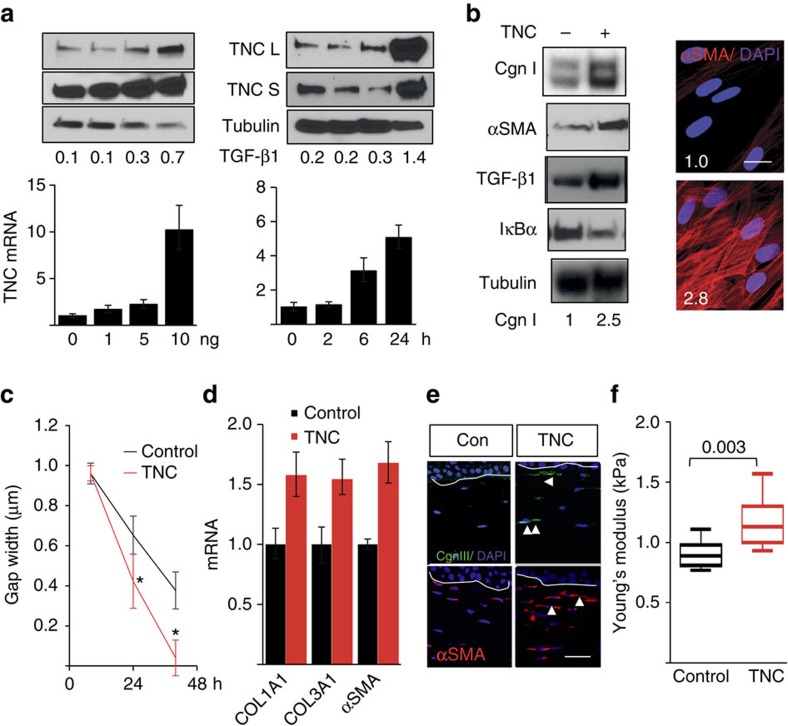
Regulation of tenascin-C expression and its effect on fibrotic responses. (**a**,**b**) Confluent foreskin fibroblasts were incubated with TGF-β (10 ng ml^−1^ or indicated concentrations) or tenascin-C (TNC) for 24 or 72 h (**b**) or indicated periods. (**a**) Whole-cell lysates, culture media and RNA were examined by western analysis (upper panels) and qPCR (lower panel). Representative immunoblots or qPCR results (means±s.e.m. of triplicate determinations). S, secreted; L, lysates. (**b**) Left panel, whole-cell lysates examined by western analysis. Cgn I, type I collagen. Representative immunoblots. Band intensities, normalized for tubulin, shown below. Right panel, immunofluorescence using antibodies to αSMA (red colour). Nuclei identified by DAPI (blue colour). Scale bar, 25 μm. Immunofluorescence intensity relative to untreated controls (means from three independent experiments) shown inside panels. (**c**) *In vitro* wound-healing assays. Fibroblasts grown in the presence or absence of tenascin-C. Results are means±s.d. of triplicate determinations in three randomly selected fields. Mann–Whitney *U*-test. (**d**–**f**) 3D organotypic raft cultures constructed including or excluding tenascin-C (2 μg ml^−1^) were incubated for 18 days. (**d**) Real-time qPCR. Results, expressed relative to GAPDH, are means±s.d. of triplicate determinations from two independent experiments. (**e**) Immunofluorescence using antibodies for collagen III and αSMA; representative images. Arrowheads indicate immunopositive cells. Scale bar, 50 μm. (**f**) Stiffness of the dermal compartment determined as described under Methods. Results represent means±s.d. from three independent experiments. Mann–Whitney *U*-test. Con, control.

To investigate the mechanisms that could potentially account for tenascin-C upregulation in SSc patients, we focused on TGF-β, which is strongly implicated in SSc pathogenesis^2^. Genome-wide expression profiling of human fibroblasts showed that TGF-β induced marked upregulation of numerous fibrotic genes, including *COMP*, *NOX4*, elastin, *PAI-1* and cadherin 2 (Supplementary Table 4 and Supplementary Fig. 2). Tenascin-C was identified as one of the top 25 TGF-β-regulated genes in fibroblasts (>2-fold increase, *P*=9.7 × 10^−8^; false discovery rate (FDR), 0.05). Dose- and time-dependent TGF-β-induced upregulation of tenascin-C in both neonatal and adult skin fibroblasts was confirmed by real-time qPCR and western analysis (Fig. 2a and Supplementary Figs 2C and 11). To identify intracellular signalling pathways underlying tenascin-C stimulation, we performed experiments using selective kinase inhibitors. The ALK5 inhibitor SB431542 completely abolished the stimulation of tenascin-C by TGF-β. Furthermore, TGF-β failed to elicit increased tenascin-C expression in Smad3^−/−^ mouse embryonic fibroblasts (MEFs), indicating that this response was Smad2/3-dependent. The PI3K inhibitor Ly294002 blocked tenascin-C stimulation induced by platelet-derived growth factor, and the MEK1/2 inhibitor U0126 had no effect (Supplementary Fig. 2B).

### Tenascin-C stimulates profibrotic responses in fibroblasts

To investigate the potential fibrotic effect of tenascin-C, we first studied fibroblasts in confluent monolayers. Incubation with recombinant tenascin-C elicited an increase in type I collagen and α smooth muscle-actin (αSMA) expression and stress fibre incorporation, accompanied by the upregulation of TLR4 (Fig. 2b and Supplementary Figs 3A and 11). Fibroblasts grown on plates coated with tenascin-C showed significantly increased rate of migration on a scratch injury compared with those grown on uncoated plastic dishes (Fig. 2c), consistent with previous reports of accelerated wound healing^12^. Moreover, fibroblast-mediated collagen gel contraction was considerably enhanced when tenascin-C was incorporated in the collagen matrix (Supplementary Fig. 3B). To examine the effect of tenascin-C on fibroblasts in a non-mechanically stressed environment, we used three-dimensional (3D) skin raft cultures constructed with collagen slurry including or excluding tenascin-C and populated with human skin fibroblasts^13^. Following incubation for up to 18 days, rafts were collected and dermal compartments were analysed. Inclusion of tenascin-C in the rafts was associated with marked increase in collagen and αSMA mRNA (Fig. 2d), enhanced type III collagen deposition and increased αSMA expression in fibroblasts resident within the dermal compartment (Fig. 2e). Furthermore, tenascin-C significantly increased the mechanical stiffness of the dermal compartment, whereas no difference was seen in stiffness between organotypic rafts constructed with or without tenascin-C in the absence of embedded fibroblasts (Fig. 2f). These complementary *ex vivo* models together demonstrate for the first time that tenascin-C elicits a broad profibrotic response in normal fibroblasts.

### Tenascin-C-induced fibrotic responses are TLR4-dependent

**Figure 3:**
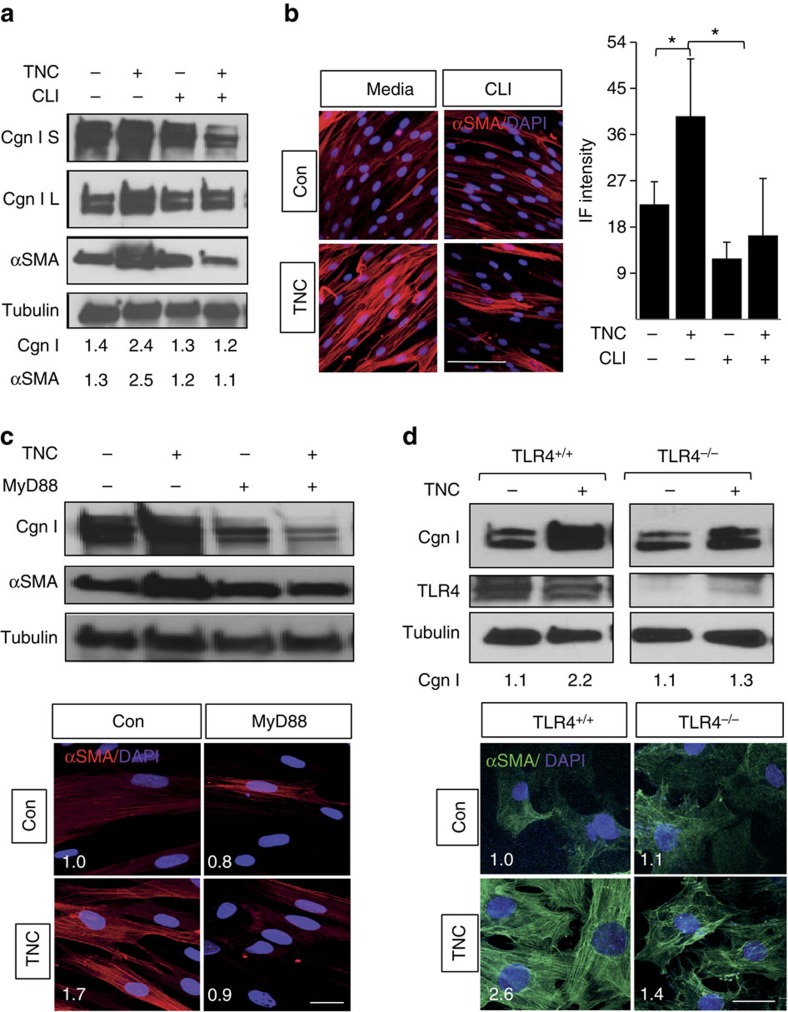
Tenascin-C-induced fibrotic responses are TLR4-dependent. (**a**–**c**) Human foreskin fibroblasts were incubated in media with tenascin-C (TNC; 2 μg ml^−1^) in the absence or presence of CLI-095 or MyD88 blocking peptide or scrambled controls for 72 h. (**a**,**c**,**d**) Whole-cell lysates analysed by western blotting. Representative immunoblots. S, secreted; L, lysates. Band intensities, normalized for tubulin, shown below. (**b**–**d**) Immunofluorescence (IF) microscopy using antibodies to αSMA, and DAPI. Representative images; scale bar, 50 μm. One way analysis of variance followed by Bonferroni's multiple comparison test. (**d**) Skin fibroblasts isolated from mice with tamoxifen-inducible fibroblast-specific TLR4 knockout (TLR4^−/−^) and control mice (that is, no tamoxifen) incubated in media with tenascin-C (2 μg ml^−1^) for 72 h. Upper panel, representative immunoblots. Lower panels, immunofluorescence; scale bar, 25 μm. Relative fluorescence intensities represent means from four randomly selected h.p.f.'s. Con, control.

As a matricellular protein with both structural as well as signalling properties, tenascin-C regulates cellular behaviour through multiple membrane receptors. In addition, tenascin-C has been shown to function as an endogenous ligand for TLR4 in macrophages, dendritic cells and synovial fibroblasts^14^,^15^. Immunoprecipitation of untreated and TGF-β-treated skin fibroblast whole-cell lysates with anti-TLR4 antibodies followed by immunoblotting revealed a direct interaction of cellular TLR4 with endogenous tenascin-C, suggesting that fibroblast responses elicited by tenascin-C might be mediated via TLR4 (Supplementary Fig. 3C). To directly assess the role of TLR4 in the profibrotic effect of tenascin-C, we pursued complementary loss-of-function approaches. First, we confirmed that tenascin-C was able to elicit classic TLR4 responses, including nuclear factor-kB activation in normal human fibroblasts (Fig. 2b). Pretreatment of cultures with a small molecule inhibitor that selectively binds to the TLR4 intracellular signalling domain^13^ completely abrogated tenascin-C-induced stimulation of collagen gene expression, and substantially reduced myofibroblast differentiation (Fig. 3 and Supplementary Figs 4A and 11). Moreover, stimulation of IL-6 and TGF-β was also abrogated. Disrupting MyD88-dependent intracellular TLR4 signalling using selective blocking peptides similarly abrogated tenascin-C-induced stimulation of collagen and αSMA expression (Fig. 3c). Endotoxin contamination of recombinant tenascin-C preparations does not account for the stimulatory responses, because preincubation of the cultures with polymyxin B (to eliminate endotoxin contamination) failed to abrogate the profibrotic effects of tenascin-C, while effectively blocking lipopolysaccharide (LPS)-induced cytokine production, as well as *COL1A1* gene expression (Supplementary Fig. 4B). Next, we examined the effects of tenascin-C in skin fibroblasts isolated from mice with fibroblast-specific TLR4 deletion. In contrast to control fibroblasts (from transgenic mice injected with corn oil) that showed a robust tenascin-C response, in TLR4-deficient skin fibroblasts tenascin-C failed to elicit stimulation of collagen synthesis or αSMA expression (Fig. 3d). These combined pharmacological and genetic approaches together establish, for the first time, a sufficient and necessary role for TLR4 in profibrotic cellular responses elicited by tenascin-C.

### Persistent fibrosis is attenuated in TNC^−/−^ null mice

**Figure 4:**
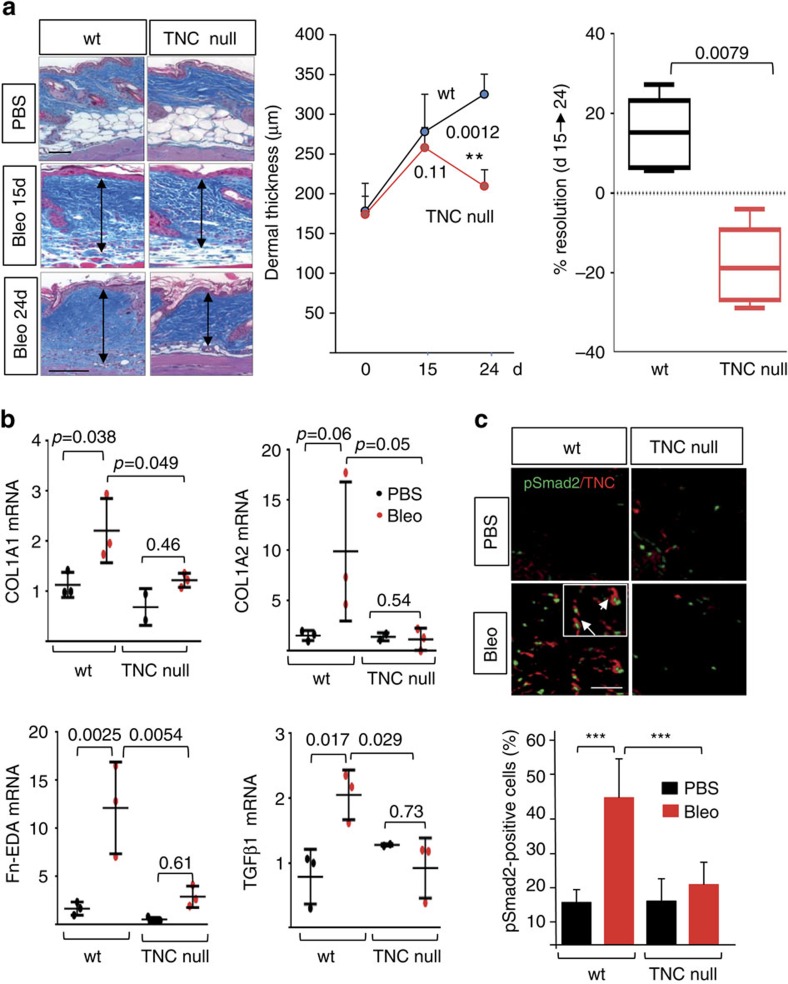
Attenuated skin fibrosis in TNC^−/−^ mice. Wild-type (WT) mice and TNC^−/−^ mice in parallel received bleomycin or PBS via s.c. injections for 10 days. Lesional skin was collected at day 15 and day 24. (**a**) Left panels, Trichrome stain. Representative images. Scale bar, 100 μm. Arrows indicate dermis. Middle panel, dermal thickness (means±s.d. of five determinations per h.p.f. from three or five mice per group). Right panel, changes in dermal thickness comparing day 24 with 15 following initial injection. Mann–Whitney *U*-test. (**b**) mRNA levels at day 24 determined by real-time qPCR. Results, normalized with GAPDH, are means±s.d. of triplicate determinations from three mice per group. One-way analysis of variance followed by Sidak's multiple comparison test. (**c**) Double immunofluorescence using antibodies to phospho-Smad2 and tenascin-C. Upper panel, representative images. Scale bar, 50 μm. Inset, higher magnification; arrows indicate co-localization of tenascin-C and nuclear phospho-Smad2. Lower panel, proportion of phospho-Smad2-positive cells within the dermis determined at four randomly selected locations per h.p.f. Results are means±s.d. from three mice per group.

Augmented tenascin-C expression in SSc, combined with its ability to induce both TLR4 and TLR4-dependent fibrotic responses, suggested a potential pathogenic role for tenascin-C in fibrosis. To explore this notion we compared experimental fibrosis in wild-type mice and TNC^−/−^ mice. Mice lacking tenascin-C are fertile and viable, although they exhibit altered behaviour and structural defects in neuromuscular junctions^16^,^17^,^18^,^19^. Bleomycin in both wild-type mice and TNC^−/−^ mice induced comparable early infiltration of F4/80-positive macrophages in the dermis, peaking at day 7. While substantial macrophage infiltration persisted at day 24 in wild-type mice, TNC^−/−^ mice showed a trend towards reduced numbers (Supplementary Fig. 5). The number of CD3-positive lymphocytes was significantly reduced in TNC^−/−^ mice at both early and late time points (Supplementary Fig. 5B and data not shown). Importantly, dermal thickness showed a progressive increase following cessation of bleomycin injections (from day 15 to 24) in wild-type mice, while TNC^−/−^ mice showed a partial resolution during the same period (Fig. 4a). Augmented expression of fibrotic genes including collagen and fibronectin-EDA (Fn-EDA), as well as prototypic TLR4 target genes such as *IL-6* and *MCP-1*, was markedly attenuated in TNC^−/−^ mice (Fig. 4b and Supplementary Fig. 6A, and data not shown). In addition, TGF-β levels, and intracellular Smad2/3 activation co-localizing with increased tenascin-C deposition within the fibrotic skin, were both diminished (Fig. 4c). Accumulation of tenascin-C was prominent in lesional skin from bleomycin-treated wild-type mice by day 4, and persisted through day 24, but was not seen in TNC^−/−^ mice (Fig. 4c and Supplementary Fig. 6B). Remarkably, enhanced fibronectin deposition in the skin was also markedly attenuated TNC^−/−^ mice (Supplementary Fig. 6C). Skin fibroblasts isolated from 4-week-old TNC^−/−^ mice showed baseline TLR4 expression that was comparable to wild-type fibroblasts. In contrast, TGF-β stimulation of type I collagen and alpha smooth muscle actin (ASMA) (protein and mRNA) was attenuated in TNC^−/−^ fibroblasts compared with identically treated wild-type fibroblasts, despite comparable levels of cellular Smad2 and Smad3 (Supplementary Fig. 7). As expected, TGF-β caused a sevenfold stimulation of tenascin-C, which was absent in TNC^−/−^ fibroblasts. Together, these results provide the first evidence that tenascin-C deficiency contributed to attenuated skin fibrosis and enhanced fibrosis resolution in bleomycin-treated mice.

**Figure 5:**
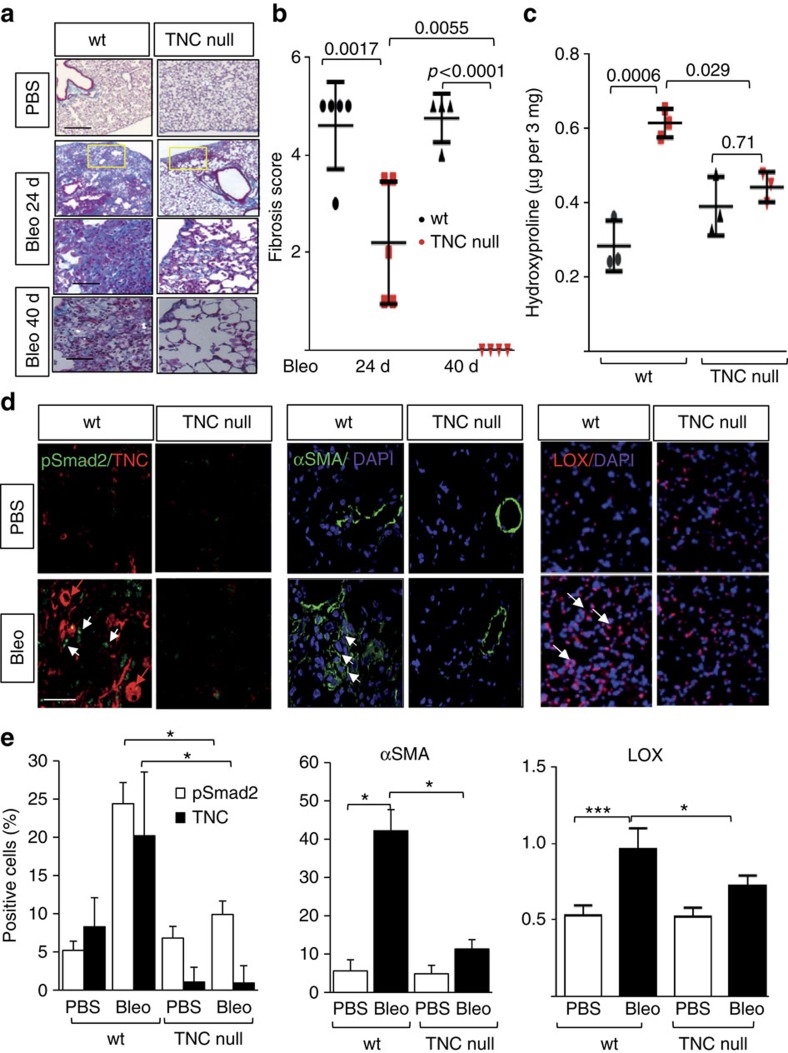
Attenuated lung fibrosis in TNC^−/−^ mice. Wild-type (WT) mice and TNC^−/−^ mice in parallel were administered bleomycin or PBS via s.c. injections for 14 days and lungs were collected 10 or 26 days following last injection. (**a**) Trichrome stain for collagen. Representative images; scale bars, 100 (top two rows) and 50 μm (bottom two rows). (**b**) Fibrosis scores (Hubner) determined in lungs from 8 h.p.f. per mice. Results are means±s.d. from four mice per group. One-way analysis of variance (ANOVA) followed by Sidak's multiple comparison test. (**c**). Collagen content. Dots represent the means±s.d. from duplicate determination from three mice per group. One-way ANOVA followed by Sidak's multiple comparison test. (**d**) Double immunofluorescence with antibodies to phospho-Smad2, tenascin-C, αSMA and LOX. Representative images. White arrows indicate immunopositive cells. Scale bars, 50 μm. (**e**) Quantitation of immunopositive cells. Results are means±s.d. from four h.p.f. per mice from three mice per group. One-way ANOVA followed by Bonferroni's multiple comparison test.

Intratracheal bleomycin administration is associated with severe acute lung injury followed by inflammation and fibrosis^20^. Bleomycin given via repeated subcutaneous (s.c.) injections also causes lung fibrosis, albeit with a more insidiously progressive course resembling that of SSc-associated lung disease^21^. To explore the role of tenascin-C in lung fibrosis, bleomycin or phosphate-buffered saline (PBS) was therefore administered to TNC^−/−^ mice and wild-type controls via s.c. injection for 14 days, and mice were killed 10 and 26 days following the last s.c. injection. Despite a reported role for tenascin-C in fetal lung branching, uninjured tenascin-C-deficient mice had lung morphology and lung mechanics similar to those in wild-type mice^22^. In wild-type mice, bleomycin elicited prominent lung changes with an influx of inflammatory cells and emergence of fibrotic foci in the subpleural region (Fig. 5a,b and Supplementary Fig. 9A). Lung fibrosis was accompanied by substantial collagen accumulation (Fig. 5c), and tenascin-C deposition within the interstitial matrix of thickened alveolar septae, whereas no tenascin-C was detectable in the lungs from TNC^−/−^ mice (Fig. 5d). Lung pathology, including pleural thickening and the numbers of fibrotic foci, was markedly ameliorated at day 24, with significantly reduced fibrosis scores, in TNC^−/−^ mice. Trichrome stain and hydroxyproline assays showed diminished accumulation of collagen. The number of αSMA-positive interstitial myofibroblasts in the lungs, as well as infiltration with macrophages and lymphocytes, were all significantly reduced in TNC^−/−^ mice (Fig. 5d,e and Supplementary Fig. 8). Bleomycin-induced lung fibrosis is dependent of canonical TGF-β signalling^23^. The proportion of phospho-Smad2-positive fibroblastic cells was substantially reduced in the absence of tenascin-C (*P*<0.05, Mann–Whitney *U*-test; Fig. 5e). By catalysing collagen and elastin crosslinking, the lysyl oxidase (LOX) family of enzymes stabilize ECM. LOX expression has been shown to be elevated in lesional SSc tissue^24^. Immunohistochemistry demonstrated that enhanced LOX expression in bleomycin-induced lung fibrosis was significantly attenuated in TNC^−/−^ mice (Fig. 5d,e). At later time point (day 40, that is, 26 days following the last bleomycin injection), TNC^−/−^ mice showed complete resolution of lung fibrosis, whereas only partial regression was found in wild-type mice (Fig. 5a,b and Supplementary Fig. 9C,D).

**Figure 6:**
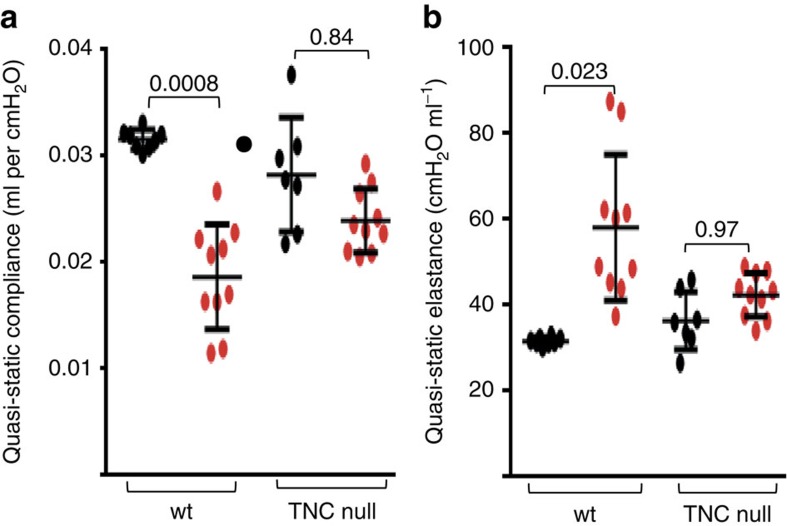
Attenuated loss of lung function in TNC^−/−^ mice. Lung mechanics determined at day 24 following initiation of s.c. bleomycin or PBS injections. (**a**) Quasi-static compliance. (**b**) Quasi-static elastance. Results are means±s.d. from 8–10 mice per group. One-way analysis of variance followed by Sidak's multiple comparison test.

Pulmonary fibrosis in SSc is associated with a restrictive ventilatory defect due to decreased lung compliance, which is determined by the stiffness of the connective tissue^25^. We hypothesized that since TNC^−/−^ lungs exhibited reduced collagen accumulation and undetectable tenascin-C, they would retain compliance in the face of bleomycin challenge. Evaluation of lung mechanics at day 24 following initiation of bleomycin or PBS showed that lung fibrosis was associated with a ∼50% decrease in quasi-static lung compliance in wild-type mice, but only nonsignificant decrease in TNC^−/−^ mice (Fig. 6). Moreover, increased quasi-static lung elastance was also significantly attenuated (*P*<0.05, Mann–Whitney *U*-test). Results of additional lung function testing are shown in Supplementary Table 5. Together, results from these morphological, biochemical and functional assays demonstrate, for the first time, that TNC^−/−^ mice are largely protected from skin and lung fibrosis.

To explore the pathogenic role of tenascin-C in non-inflammatory skin fibrosis, we generated *TNC*^−/−^*;Tsk/*+ mice. *Tsk/+* mice develop skin fibrosis caused by a duplication mutation in *FBN1* in the absence of inflammation and vascular changes^26^. By 12 weeks of age, *Tsk/*+ mice displayed a substantial increase in hypodermal thickness, collagen gene expression and tenascin-C accumulation in the subcutaneous loose connective tissue layer of the skin (Supplementary Fig. 10). In comparison, *Tsk/+* mice lacking tenascin-C showed attenuated spontaneous hypodermal thickening (Supplementary Fig. 10A). Moreover, collagen and tenascin-C deposition in the hypodermis and αSMA accumulation were reduced, indicating for the first time that tenascin-C plays a role in the development of hypodermal fibrosis in the *Tsk/+* mouse model of scleroderma.

## Discussion

While much has been learned about the triggers initiating fibroblast activation, the factors responsible for maintaining lesional fibroblasts in a persistently activated state in SSc remain largely unknown^2^. Dissecting the pathogenic networks that underlies self-sustaining fibroblast activation could provide insight into chronic fibrosis, and reveal vital targets for anti-fibrotic therapies aimed at halting progression and promoting regression of fibrosis^27^. Genetic and genomic studies highlight the robust association of SSc with inflammation, innate immunity and TLR signalling^3^,^28^. In particular, the expression of both TLR4 and its co-receptor CD14 is elevated in SSc lesional skin, and levels show correlation with disease progression^29^,^30^. Moreover, endogenous TLR4 ligands show persistent accumulation within lesional tissues^13^. These observations suggest the following plausible scenario: environmental exposure-induced upregulation of TLR4 and its endogenous ligands in a genetically susceptible host elicits persistent TLR4 signalling, which amplifies and sustains the process of fibrosis. The present studies indicate that within lesional SSc microenvironments, tenascin-C functions as a ‘damage-associated molecular pattern' that is recognized as a TLR4 ligand and drives TLR4-dependent fibroblast activation underlying persistence of tissue fibrosis.

While TLRs on monocytes, macrophages, dendritic cells and other cells of the immune system are recognized for their fundamental roles in antimicrobial responses, there is growing appreciation for the pathogenic role of non-immune cell TLR signalling in chronic inflammatory and fibrotic processes^31^,^32^. By gene expression profiling using the prototypic TLR4 agonist LPS, we showed that in contrast to its proinflammatory effect on macrophages, in skin fibroblasts a predominantly profibrotic response was induced, with upregulation of genes implicated in tissue remodelling and wound healing^29^. In these cells TLR4 ligands caused MyD88-dependent suppression of anti-fibrotic microRNAs (miR29a and miR29b) combined with downregulation of the TGF-β pseudo-receptor BAMBI, resulting in augmented TGF-β signalling intensity^31^. Immunomodulatory molecules implicated in driving aberrant TLR signalling in SSc include viruses such as cytomegalovirus, parvovirus B19, Epstein-Barr virus and the environmental fungus *Rhodotorula glutinis*^33^,^34^, as well as autoantibodies capable of binding to, and activating, TLR4 (ref. 35). Of particular interest in this regard are host-derived ‘sterile' TLR4 ligands driving disease persistence and progression^5^,^31^,^36^. Endogenous TLR4 ligands represent a structurally diverse group of intracellular and extracellular proteins, peptides, proteoglycans, phospholipids and nucleic acids that enable the host to mount an innate immune response designed to clear debris and repair tissue injury^37^,^38^. Collectively termed damage-associated molecular patterns, these endogenous signals are normally sequestered from and inaccessible to TLRs and related pattern recognition receptors. On cell injury or cell death, they are released from cells, or emerge via extracellular modifications such as enzymatic cleavage, or are produced *de novo*, and are recognized by and trigger the activation of TLRs and related pattern recognition receptors. Particularly relevant as endogenous TLR ligands in the context of fibrosis are the ECM glycoproteins fibronectin-EDA and tenascin-C.

To characterize DAMPs potentially involved in SSc pathogenesis and disease progression, we carried out a survey of the expression of endogenous putative TLR4 ligands in the lesional skin. Of several candidates, Fn-EDA, an alternately spliced fibronectin isoform, and tenascin-C appeared to be most highly upregulated. We showed previously that Fn-EDA is aberrantly deposited in both lesional skin and lungs from SSc patients, and elicits TLR4-dependent fibrotic responses^13^. The tenascins represent a family of pleiotropic ECM glycoproteins with both structural and signalling functions^6^,^7^. Expression of tenascin-C is prominent during embryogenesis, negligible in healthy skin, and transiently elevated in wound healing, where it orchestrates self-limited tissue repair^6^. Tenascin-C consists of four distinct domains that interact with ECM constituents, soluble factors and cell surface molecules including integrins and TLRs^6^. The tenascin-C gene is subject to extensive alternate splicing, and individual tenascin-C variants may elicits diverse biological responses^6^. In particular, the large tenascin-C variants have been linked with reduced focal adhesion activity and enhanced cell migration and matrix degradation, processes that favour cancer progression. However, the distinct biological roles of various tenascin-C isoforms in non-malignant processes including fibrosis still remain largely unknown^6^.

Tenascin-C has potent effects on macrophage activation and stromal cell proliferation, migration and ECM assembly, consistent with a key role in tissue remodelling and wound healing^7^. Persistent tissue expression of tenascin-C is a pathological hallmark of cancer and chronic inflammation. Altered tenascin-C expression or function are linked with fibrosis in animal models^39^. For instance, experimental acute lung injury, myocardial infarction, liver and corneal injury were all shown to be accompanied by increased tenascin-C accumulation, and attenuation of consequent fibrosis in TNC^−/−^ mice^40^,^41^,^42^,^43^. In humans, both idiopathic and SSc-associated forms of pulmonary fibrosis are accompanied by elevated tenascin-C^11^,^44^. In the skin tenascin-C shows transient elevation during wound healing, while keloid lesions are associated with sustained upregulation^45^.

Our immunofluorescence surveys identified tenascin-C as one of the most highly upregulated matricellular proteins in SSc skin biopsies, and its levels showed significant correlation with the skin score. Levels of tenascin-C mRNA were also elevated and correlated with both TLR4 and IL-6 expression in the same biopsies. Moreover, fibroblasts isolated from SSc biopsies showed constitutive tenascin-C production in culture, indicating that increased tenascin-C accumulation might at least in part result from its cell autonomous overproduction by activated fibroblasts. Cell-autonomous tenascin-C upregulation by skin and lung fibroblasts has been noted in various forms of fibrosis, possibly due to autocrine stimulation by TGF-β^11^,^44^. In SSc patients, circulating tenascin-C was elevated in both early- and late-stage disease. Increased tenascin-C in skin from patients with long-standing SSc indicates persistent upregulation of tenascin-C expression, which might play a key role in maintaining, rather than initiating, of tissue fibrosis. During dynamic processes of skin remodelling, wound healing and fibrosis, matrix accumulation and deposition are regulated by a network of matricellular proteins, including fibronectin, SPARC, CCN2, as well as tenascin-C, many of which are directly regulated by TGF-β^46^. Tenascin-C interacts with many of these matricellular proteins, and thereby modulates both fibrillogenesis and matrix organization. The present results indicate that persistent tenascin-C deposition contributes to the pathological persistence of skin and lung fibrosis, and is along with TGF-β, CCN2 and others, a major factor in the process.

While tenascin-C is known to engage surface molecules, including αvβ3 and αvβ6 surface integrins, to elicit cell-type and context-specific responses^47^,^48^, a role for surface TLRs in mediating tenascin-C effects has also been described. In particular, TLR4 has been implicated in driving tenascin-C-dependent inflammation in models of synovitis^14^, cerebral vasoconstriction^49^, hepatic ischemia reperfusion injury^50^, and adipocyte activation and foam cell formation^51^. The present results add to this list by indicating that tenascin-C elicits TLR4-dependent fibrotic responses, including enhanced synthesis of collagens, myofibroblast transition, fibroblast migration and contractility, and secretion of IL-6 and TGF-β, as well as upregulation of TLR4. Multiple complementary strategies confirmed that these profibrotic effects of tenascin-C were mediated via TLR4. It is noteworthy that tenascin-C expression levels in individual SSc skin biopsies showed a positive correlation with TLR4 as well as with IL-6, and in skin fibroblasts tenascin-C elicited TLR4-dependent fibrotic responses, including upregulation of IL-6, and of TLR4 itself. TLR4 signalling within lesional tissue promotes tenascin-C production and accumulation, and tenascin-C in turn itself drives both TLR4 expression and activity, creating a feed-forward loop driven by persistently elevated tenascin-C.

Mice with targeted deletion of tenascin-C display altered inflammatory and fibrotic responses^7^. Arthritis progression was attenuated in TNC^−/−^mice, and protection was associated with reduced secretion of inflammatory cytokines^14^. At sites of tissue injury, tenascin-C provides a scaffold for immune cell migration and adhesion^52^. While skin wounds were reported to heal normally in TNC^−/−^mice^53^, other studies show attenuation of fibrosis in the kidneys^16^, cornea^43^, heart^54^, liver^42^ and lungs^40^. Moreover, genetic variants at the tenascin-C locus are associated with increased risk of Achilles tendon injury^55^. The present studies showed that bleomycin-induced skin and lung fibrosis, and spontaneous hypodermal fibrosis in the *Tsk/+* mouse, were ameliorated in the absence of tenascin-C, and TNC^−/−^ mice showed accelerated fibrosis resolution. Isolated fibroblasts lacking tenascin-C show dampened fibrotic responses in culture. While cell-autonomous attenuation of fibrotic responses in TNC^−/−^ fibroblasts has been previously linked to impaired Smad3 levels^40^, we and others found that TNC^−/−^ fibroblasts had Smad2/3 levels that were comparable to wild-type fibroblasts, and showed intact Smad2/3 activation, as well as unaltered TLR4 expression^43^. Regardless of the precise underlying mechanisms, the cell-autonomous defect in fibrotic signalling in TNC^−/−^ fibroblasts uncovered here might contribute to enhanced resolution of fibrosis in TNC^−/−^ mice with two complementary models. We propose that reduced TLR4 signalling in TNC^−/−^ mice is responsible for attenuated fibrosis and accelerated resolution. Tenascin-C interacts directly with, and stimulates the production of fibronectin and its incorporation into the matrix^56^. While fibronectin is a potent TLR4 agonist, in TNC^−/−^ mice it might be insufficient to compensate for the loss of tenascin-C as an endogenous TLR4 ligand, since we found that fibronectin expression is itself reduced in lesional skin, as reported previously with other injury models^16^,^43^,^53^,^57^.

The present results indicate that patients with SSc appear to lose tight spatiotemporal control of tenascin-C production and accumulation. In mice treated with bleomycin, rapid induction of tenascin-C is followed by its persistence in lesional tissue, while mice lacking tenascin-C show reduced collagen and fibronectin deposition and attenuated TLR4 signalling with accelerated resolution of tissue inflammation and fibrosis. These findings lend support for the notion that pathological tissue fibrosis SSc is perpetuated via a TLR4-dependent amplification loop that is driven by damage-associated endogenous immunostimulatory molecules such as tenascin-C generated within injured microenvironments. These observations are broadly congruent with the emerging ‘DAMP hypothesis' implicating endogenous TLR agonists in chronic inflammatory and autoimmune processes as well as in cancer, ageing and fibrosis^5^,^37^,^58^. Strategies to selectively target the generation and accumulation of tenascin-C and other pathogenic DAMPs, or their recognition by TLR4 and co-receptors, or block intracellular signalling pathways downstream of activated TLR4, might yield effective therapies for the control of fibrosis. However, DAMPs also have vital protective functions required for tissue repair following injury, such as repair of acute aortic aneurysms by deposition of tenascin-C within the injured vascular wall^59^. Accordingly, caution will be needed in the pursuit of therapeutic approaches that target TLR4 activation by its endogenous ligands, and much remains still to be learned regarding the optimal dosing and timing of such interventions in SSc.

## Methods

### Patient data

A total of 187 subjects with SSc and 63 healthy volunteers were recruited from the scleroderma clinics of Northwestern University Feinberg School of Medicine, University of Pittsburgh School of Medicine and Boston University School of Medicine (Tables 1, 2, 3, 4). All patients fulfilled American College of Rheumatology (ACR) criteria for the classification of SSc with informed consent^60^. Information obtained at the time of tissue or serum collection included demographics, disease duration (defined as interval between the first non-Raynaud SSc event and sampling), MRSS (range 0–51) and autoantibody testing. On the basis of disease duration, patients were subclassified into early (<24 months)- or late (>24 months)-stage disease subsets. Pulmonary function tests and high-resolution computed tomography of the chest were performed within 6 months of serum collection. Exclusion criteria included inability to provide informed consent and the information obtained from the patients as described.

### Cell culture and reagents

Fibroblast cultures were established by explanation from neonatal foreskin, or from skin biopsies of the clinically affected forearms of patients with SSc and forearms of healthy adult volunteers^61^. Cultures of mouse embryonic fibroblasts from Smad3^−/−^ or wild-type mouse embryos were established. Low-passage fibroblasts (*P*<5) were studied at early confluence in monolayers on plastic dishes, or were embedded in rat type I collagen used to construct 3D organotypic raft cultures^61^,^62^. To generate *TLR4*^−/−^ fibroblasts, mice carrying tamoxifen-inducible Cre (*Cre-ERT*) under the control of a fibroblast-specific Col1a2 enhancer were crossed with mice homozygous for the floxed TLR4 allele (TLR4^fl/fl^ mice were a generous gift from Dr Christopher Karp from Cincinnati Childrens Hospital; COL1A2-*Cre-ERT* from The Jackson Laboratory, Bar Harbor, ME) to generate *COL1A2-Cre*^*+/−*^*;TLR4*^*flox/flox*^ homozygous mice. At 3 weeks of age, female *COL1A2-Cre*^*+/−*^*;TLR4*^*flox/flox*^ mice were injected with tamoxifen (20 μg, Sigma) or vehicle (corn oil) intraperitoneally. Skin was collected 21 days later, and fibroblast lines were established by explantation. Cultures were maintained in Dulbecco's modified Eagle's medium (DMEM) supplemented with 10% fetal calf serum (FCS; Gibco BRL, Grand Island, NY), 1% vitamin solutions and 2 mM L-glutamine. All other tissue culture reagents were from Lonza (Basel, Switzerland). For experiments, fibroblasts were placed in serum-free media containing 0.1% bovine serum albumin (BSA) for 24 h before addition of TGF-β1 (PeproTech, Rocky Hill, NJ), or recombinant human tenascin-C (Millipore, Catalogue CC0651, Cambridge, MA) purified from human U251glioma cell line (migrating around 270–300 kDa, data not shown). In selected experiments, cultures were pretreated for 60 min with the TLR4 inhibitor CLI-095, or the MyD88 inhibitor Pepinh-MYD or Pepinh-control (10 μM; both from InvivoGen, San Diego, CA), or polymyxin B (ThermoFisher Scientific, Grand Island, NY).

### 3D human skin equivalents

To evaluate the fibroblast-modulating effects of tenascin-C in a physiologically relevant environment, 3D organotypic raft cultures that recapitulate the topography and biochemical properties of human skin were constructed^61^. Briefly, foreskin fibroblasts (300,000 cells per ml) were resuspended in a slurry of rat tail type I collagen (4 mg ml^−1^, from BD Biosciences, San Jose, CA) with or without tenascin-C (2 μg ml^−1^, Millipore) and seeded in 12-well plates (1.5 ml per well)^13^. Plugs were allowed to polymerize and following overnight incubation in media seeded with foreskin epidermal keratinocytes (200,000 cells per ml) isolated from a pool (*n*=3) of normal skin biopsies. Three days later the skin rafts were transferred to 60-mm tissue culture dishes, exposed to air to promote epidermal stratification and incubated in media with epidermal growth factor (5 ng ml^−1^) for up to 18 days. Rafts were then collected, fixed in 10% neutral buffered formalin and embedded in paraffin for immunofluorescence staining. Substrate stiffness of the raft dermal compartment was determined using a parallel-plate rheometer^13^, and Young's modulus was calculated using US 200 software (Oscillatory Shear Paar Physica MCR Rheometer, Anton Paar GmbH, Houston, TX).

### Cell migration and collagen gel contraction assays

The effects of tenascin-C on fibroblast function were further evaluated by *in vitro* wound-healing and collagen gel contraction assays. Briefly, human skin fibroblasts were seeded on tenascin-C-coated plates in serum-free DMEM, and confluent monolayers were mechanically wounded using p1000 pipette tips. Following incubation of the cultures for indicated periods, wound gap widths (μm) were determined at six randomly selected sites per high power field (h.p.f.). Collagen gel contraction assays were performed with normal skin fibroblasts seeded in type I collagen gels^13^. After incubation of the gels in medium containing tenascin-C (2 μg ml^−1^) for the indicated intervals, gel diameters were determined with ImageJ software (National Institutes of Health (NIH), Bethesda, MD). All experiments were performed in triplicate.

### Isolation and analysis of RNA

Total RNA isolated from fibroblasts cultures, 3D organotypic raft cultures and human and mouse skin biopsies was reverse-transcribed to cDNA using Supermix and analysed by real-time qPCR^13^,^29^. The products (20 ng) were amplified using SYBR Green PCR Master Mix (Applied Biosystems) on an Applied Biosystems 7500 Prism Sequence Detection System. The results were normalized to GAPDH RNA levels, and fold change in samples was calculated^13^.

### Genome-wide expression profiling and data analysis

To examine fibroblast-modulating effects of TGF-β at the genome-wide level, confluent cultures were incubated in serum-free media with TGF-β for 24 h, and RNA was isolated using RNeasy minikits (Qiagen, Valencia, CA). The integrity of RNA was ascertained using an Agilent Bioanalyzer (Agilent Technologies, Santa Clara, CA), and cDNA was labelled using Ambion labelling kits (Ambion) and hybridized to Illumina human HT12 v4 Expression Microarray Chips (Illumina, San Diego, CA), as previously described^29^. Raw signal intensities for each probe were obtained using Illumina Bead 2 studio data analysis software and imported to the Bioconductor lumi package for data transformation and normalization^29^. Probes with all samples near or lower than background levels were filtered. To control for multiple testing and reduce the false-positive rate (FDR), stringent statistical criteria were used to identify differentially expressed genes with raw *P*<0.01 and FDR-adjusted *P*<0.05.

### Western and co-immunoprecipitation/immunoblot analyses

At the end of the experiments, fibroblasts were collected, culture supernatants and whole-cell lysates were prepared and equal amounts of proteins (10–20 μg per lane) subjected to western analysis using primary antibodies specific for human type I collagen (Southern Biotechnology, 1:800, Birmingham, AL), tenascin-C (Novus Biologicals, 4C8Ms catalogue # NB110-68136, Littleton, CO; detects alternately spliced isoforms of tenascin-C including FN III-B domain, 1:1000), tubulin (Sigma-Aldrich, St Louis, MO) and phospho-Smad2 (Cell Signaling, Ser465/467, 1:1000). Membranes were then incubated with appropriate secondary antibodies and subjected to enhanced chemiluminescence detection using ECL Reagent (Pierce, Rockford, IL). In selected experiments, whole-cell lysates (∼500 μg) were immunoprecipitated with antibodies to TLR4 (Santa Cruz, H80, 1:250, Dallas, TX), separated by gel electrophoresis and immunoblotted using antibodies to tenascin-C (Abcam, 4C8Ms, 1:1,000, Cambridge, MA) or TLR4.

### Immunofluorescence confocal cytochemistry

Fibroblasts seeded on 8-well Lab-Tek II chamber glass slides (Nalgene Nunc International, Naperville, IL) were incubated in serum-free DMEM with or without tenascin-C (2 μg/ml) for up to 72 h. Cells were then fixed, permeabilized, and incubated with antibodies to αSMA (Sigma, St Louis, MO) at 1:100 or 1:500 dilution, followed by Alexa-fluor-labelled secondary antibodies (Invitrogen, 1:3000). Nuclei were identified using 4,6-diamidino-2-phenylindone (DAPI), and immunofluorescence was evaluated under a Zeiss UV Meta 510 confocal microscope (Carl Zeiss Inc, Jena, Germany)^29^ and Nikon C2.

### Immunofluorescence evaluation of tenascin-C levels in skin biopsies

To determine tenascin-C levels in tissue, we examined forearm skin biopsies (60% classified as inflammatory and 40% diffuse proliferative) from 18 SSc patients and 9 healthy adults (Cohort 2) by immunofluorescence^13^. Paraffin-embedded sections (4 μm) were incubated with primary mouse monoclonal antibodies to tenascin-C (Abcam, T2H5, catalogue# 3970) at a dilution of 1:50. These antibodies recognize the large (265 kDa) tenascin-C isoform. Additional primary antibodies to αSMA (Abcam, 1:100), fibronectin-EDA (1:100, IST-9, Abcam) and TGF-β1 (Santa Cruz, 1:100) were used, followed by mouse Alexa-fluor secondary antibodies (Invitrogen) or DAPI. Imunofluorescence was evaluated under a Zeiss UV Meta 510 confocal microscope, and computer-generated 3D plots of fluorescence intensity were analysed using Image J.

### Measurement of circulating tenascin-C levels

Levels of circulating tenascin-C were determined in a discovery cohort of 50 SSc patients and 26 age-matched healthy controls (Cohort 3). The Human Tenascin-C Large (FN III-C) Assay Kits (IBL, Gunma, Japan) used detects the alternately spliced isoform of tenascin-C that includes the FN III-C domain. Results were calculated from standard curves (range, 24–0.38 ng ml^−1^). The coefficient of variation from replicates in all analyses was 2.9% (mean). Tenascin-C levels were also determined in an independent replication cohort of 62 SSc patients (50% with ILD by chest radiography or high-resolution computed tomography) and 10 age-matched healthy controls (Cohort 4). In a third study cohort comprised of 38 SSc patients (76% female, median disease duration 25.5 months) and 22 age-matched healthy controls (cohort 5), serum levels of tenascin-C were determined by enzyme-linked immunosorbent assay (Myriad Rules Based Medicine, Austin, TX).

### Experimental model of skin and lung fibrosis

Complementary mouse models of fibrosis were employed to evaluate the role of tenascin-C *in vivo*. First, 8-week-old female *TNC*^−/−^ mice (C57BL/6 N-TgH, from RIKEN, Japan) and C57BL/6J mice (The Jackson Laboratory) in parallel received bleomycin (10 mg kg^−1^ per day) or PBS daily via s.c. injections for 10 days, and killed at various time points for up to 40 days after the last injection. All treatment groups consisted of at least five mice and experiments were repeated three times with consistent results. Cultures of fibroblasts were established by explantation from dorsal skin from wild-type and *TNC*^−/−^ mice. In a complementary non-inflammatory model of fibrosis, *Tsk/+* mice (C57BL/6 background, The Jackson Laboratory) at 8 weeks of age were crossed with *TNC*^−/−^ mice to generate *TNC*^−/−^*;Tsk/*+ mice. At 12 weeks of age, female *TNC*^−/−^*;Tsk/*+ mice and C57BL/6J control mice were killed, and lesional skin and lungs were collected for analysis^21^.

Four-micometre-thick sections of paraffin-embedded tissues were stained with haematoxylin and eosin or Trichrome. Thickness of the dermis and hypodermis, defined as the distance from the epidermal–dermal junctions to the dermal–adipose junction or to the loose connective tissue subjacent to the panniculus carnosus, respectively, were determined at five randomly selected sites per h.p.f. Sections of lungs stained with haematoxylin and eosin were scored for fibrosis^63^ in a blinded manner by an expert pulmonary pathologist (K.R.). For immunofluorescence analyses, paraffin-embedded skin and lung sections were incubated with primary rabbit antibodies against αSMA (1:100), tenascin-C (T2H5, 1:500), LOX (100) or phospho-Smad2 (Cell Signaling, 1:100), followed by Alexa-fluor-labelled rabbit secondary antibodies. Nuclei were detected using DAPI. Slides were evaluated under a Zeiss UV Meta 510 confocal microscope. For immunohistochemistry, sections of paraffin-embedded skin and lungs were immunolabelled with primary rabbit antibodies against F4/80 (1:500, eBioscience, San Diego, CA) and CD3 (1:3000, Abcam), followed by appropriate biotinylated secondary antibodies (1:250, all from Jackson Immunoresearch, West Grove, PA) and detected using biotin complex conjugated with horseradish peroxidase (Vector Laboratories, Burlingame, CA) and DAB for colour development (Dako, Carpinteria, CA). Collagen content of the lungs was determined by hydroxyproline assays (Colorimetric Assay Kits, Biovision, Milpitas, CA).

### Evaluation of lung mechanics

To evaluate the impact of tenascin-C on bleomycin-induced functional changes in lung mechanics, 9- to 10-week-old wild-type female mice and *TNC*^−/−^ mice treated with PBS or bleomycin were mechanically ventilated on a computer-controlled piston ventilator (FlexiVent mouse ventilator (ScireQ, Montreal, Canada), as described^64^,^65^. A standard ventilation history for each mouse was obtained with three total lung capacity maneuvers before the forced oscillation and quasi-static pressure–volume curve protocols that were used to calculate airway resistance, dynamic and quasi-static tissue compliance, dynamic and quasi-static tissue elastance and additional parameters of respiratory function.

### Statistical analysis

For comparison between healthy subjects and subjects with SSc, sample sizes were chosen to achieve 80% power to detect an effect size of 0.69 between the two groups with a significance level of 0.05 using a two-sided two-sample *t*-test. The normality of distribution of serum tenascin-C levels was determined by Kolmogorov–Smirnov test. Mann–Whitney *U*-test was used to compare tenascin-C levels. Spearman's rank and Pearson's correlations were calculated to measure the correlation between tenascin -C (TN-C) levels and clinical parameters. Data were analysed using SPSS Statistics 17 (Chicago, IL) and SAS 9.4 (Cary, NC). A *P* value <0.05 was considered statistically significant. Data are presented as means±s.d. unless otherwise indicated. For *in vitro* and *in vivo* experiments, Mann–Whitney *U*-test and Student's t-test were used for comparisons between two groups, with a *P* value <0.05 considered statistically significant. Comparisons among three or more groups were performed using analysis of variance followed by Bonferroni or Sidak's correction for multiple comparisons. Data were analysed using Graph Pad prism (Graph Pad Software version 6, Graph Pad Software Inc., CA).

### Study approval

Human studies were approved by the Institutional Review Boards of Northwestern University, Boston University and the University of Pittsburgh. All participants provided written informed consent. Animal studies were conducted in accordance with NIH guidelines for the care and use of laboratory animals, and under protocols approved by the Northwestern University IACUC.

## Additional information

**Accession codes:** The microarray data have been deposited in GEO under accession code GSE79621.

**How to cite this article:** Bhattacharyya, S. *et al*. Tenascin-C drives persistence of organ fibrosis. *Nat. Commun.* 7:11703 doi: 10.1038/ncomms11703 (2016).

## Supplementary Material

Supplementary Information Supplementary Figures 1-11 and Supplementary Tables 1-6.

## Figures and Tables

**Table 1:** Clinical characteristics of Cohorts 1 and 2.

Subject code	Disease stage	Age	Gender	Subtype	MRSS	SSc intrinsic subset assignment
Cohort 1						
ssc01LF	Late	51	F	dcSSc	36	Inflammatory
ssc02B LF	Late	46	F	dcSSc	35	Inflammatory
ssc05 BRA	Early	40	F	dcSSc	32	Inflammatory
ssc06BRA	Early	54	F	lcSSc	16	Inflammatory
ssc08BLA	Early	65	F	dcSSc	12	Inflammatory
ssc10BLF	Early	52	F	dcSSc	13	Inflammatory
ssc1004BLA	Late	27	F	dcSSc	26	Normal-Like
ssc1156BLA	Early	48	F	SSc/PM	20	Inflammatory
ssc1213LA	Late	30	F	dcSSc	4	Inflammatory
ssc1002BLA	Late	34	F	dcSSc	32	Fibroproliferative
ssc1103 BLA	Late	54	F	dcSSc	13	Inflammatory
ssc1215LA	Late	30	F	dcSSc	4	Inflammatory
ssc1269LA	Early	50	F	dcSSc	14	Inflammatory
						
Cohort 2						
SScMH_03_Base_LA	Early	48	F	dcSSc	21	Inflammatory
SScMH_04_Base_LA	Early	45	F	dcSSc	9	Normal-like
SScMH_05_Base_RA	Early	40	F	dcSSc	32	Inflammatory
SScMH_06_Base_RA	Early	54	F	lcSSc	16	Inflammatory
SScMH_08_Base_LA	Early	65	F	dcSSc	12	Inflammatory
SScMH_12_Base_LA	Early	51	F	dcSSc	14	Fibroproliferative
SScMH_13_Base_LA	Early	57	F	dcSSc	17	Inflammatory
SScMH_17_Base_LA	Early	53	M	dcSSc	35	Inflammatory
SScMH_18_Base_LA	Late	57	F	dcSSc	11	Fibroproliferative
SScMH_27_Base_LA	Early	47	F	dcSSc	29	Inflammatory
SScMH_30_Base_LA	Early	56	F	dcSSc	17	Fibroproliferative
SScMH_31_Base_LA	Early	56	F	dcSSc	15	Inflammatory
SScMH_42_Base_LA	Early	47	M	dcSSc	16	Inflammatory
SScReg_1103_Base_LA	Late	54	F	dcSSc	13	Inflammatory
SScMH_13_Base_LA	Early	57	F	dcSSc	17	Inflammatory
SScMH_30_Base_LA	Early	56	F	dcSSc	17	Fibroproliferative
SScMH_44__LA	Early	68	F	dcSSc	12	Fibroproliferative
SScMH_26_Base_LA	Early	58	F	dcSSc	18	Fibroproliferative

SSc subjects providing skin biopsy samples (lesional forearm) for RNA and immunofluorescence analysis. dcSSc, diffuse cutaneous SSc; lcSSc, limited cutaneous SSc; PM, polymyositis overlap; M, male, F, female. *Early, disease duration <2 years from first non-Raynaud disease manifestation; late, >2 years from first non-Raynaud manifestation. MRSS, modified Rodnan skin score (1 to 51). Intrinsic subsets: D, diffuse, inflammatory. For Cohort 1, controls were healthy volunteers (100% female; median age, 47 years; range, 27 to 65 years). For cohort 2, controls were healthy subjects (90% female; median age, 56 years; range, 47 to 68 years).

**Table 2:** Clinical characteristics of Cohort 3.

Subject code	Disease stage	Age	Gender	Subtype	MRSS	SSc intrinsic subset assignment
SScReg_1146	Late	41	F	dcSSc	27	NA
SScReg_1165	Early	40	F	dcSSc	45	NA
SScReg_1271	Late	50	F	dcSSc	25	Normal-like
SScReg_1017	Late	41	F	dcSSc	21	Inflammatory
SScReg_1332	Late	47	F	dcSSc	26	NA
SScReg_1327	Early	48	F	dcSSc	26	NA
SScReg_1119	Early	56	M	dcSSc	38	NA
SScReg_1046	Late	40	F	dcSSc	16	NA
SScReg_1580	Early	56	M	dcSSc	34	NA
SScReg_1074	Late	60	F	dcSSc	2	NA
SScReg_1124	Late	64	M	lcSSc	4	NA
SScReg_1235	Late	67	F	lcSSc	2	NA
SScReg_1283	Early	61	F	lcSSc	4	NA
SScReg_1284	Late	40	F	lcSSc	3	NA
SScReg_1307	Late	36	M	lcSSc	4	NA
SScReg_1364	Late	55	M	lcSSc	4	NA
SScReg_1452	Early	51	M	lcSSc	4	NA
SScReg_1105	Late	60	F	lcSSc	2	NA
SScReg_1297	Late	32	F	dcSSc	3	NA
SScReg_1185	Early	58	F	dcSSc	25	NA
SScReg_1248	Late	63	F	dcSSc	36	Inflammatory
SScReg_1298	Early	46	F	dcSSc	23	NA
SScReg_1354	Early	43	F	dcSSc	34	NA
SScReg_1426	Late	28	F	dcSSc	18	NA
SScReg_1696	Early	51	M	dcSSc	48	Inflammatory
SScReg_1062	Early	48	F	dcSSc	19	Inflammatory
SScReg_1106	Early	31	F	lcSSc	2	NA
SScReg_1194	Early	39	F	lcSSc	2	NA
SScReg_1109	Late	55	F	lcSSc	4	NA
SScReg_1295	Early	60	F	lcSSc	3	NA
SScReg_1303	Early	45	F	lcSSc	3	Inflammatory
SScReg_1318	Early	60	F	lcSSc	2	NA
SScReg_1014	Late	49	F	lcSSc	4	NA
SScReg_1132	Early	47	F	lcSSc	2	NA
SScReg_1142	Late	75	F	lcSSc	2	NA
SScReg_1428	Late	51	F	lcSSc	4	NA
SScReg_1527	Late	54	F	lcSSc	3	NA
SScReg_1596	Late	62	F	lcSSc	2	NA
SScReg_1153	Late	34	F	lcSSc	2	NA
SScReg_1245	Late	55	F	lcSSc	2	NA
SScReg_1224	Early	54	F	dcSSc	11	NA
SScReg_1300	Early	43	F	lcSSc	12	Inflammatory
SScReg_1369	Early	47	F	lcSSc	11	NA
SScReg_1494	Early	58	F	dcSSc	10	Inflammatory
SScReg_1734	Late	33	F	lcSSc	10	NA
SScReg_1174	Late	50	F	lcSSc	5	NA
SScReg_1373	Late	29	F	dcSSc	14	NA
SScReg_1070	Late	62	F	dcSSc	13	NA
SScReg_1571	Early	66	F	dcSSc	13	NA
SScReg_1150	Late	52	F	dcSSc	10	NA

F, female; M, male; NA, not applicable.

SSc subjects providing serum samples for determination of tenascin-C levels (discovery cohort). Early, <2 years from first non-Raynaud disease manifestation; late, >2 years from first non-Raynaud manifestation. MRSS, 1–51. Intrinsic subsets^8^,^9^: D, diffuse, I, inflammatory. Controls were healthy subjects (88% female; median age, 50 years; range, 28–75 years).

**Table 3:** Comparison of SSc patients (Cohort 3) with low versus high serum tenascin-C levels.

Clinical feature	TNC<105.4 ng ml^−1^, *N*=39	TNC>105.4 ng ml^−1^, *N*=11	*P* value
Subtype (% dcSSc)	41%	82%	*P*=0.037 (Fischer exact test)
MRSS	4 (2–14)	25 (11–36)	*P*=0.006 (Mann–Whitney *U*)
			
Autoantibodies			
ATA	26% (8/31)	22% (2/9)	*P*=1.00 (Fischer exact test)
ACA	29% (9/31)	11% (1/9)	*P*=0.404 (Fischer exact test)
ARA	10% (3/31)	11% (1/9)	*P*=1.00 (Fischer exact test)
Disease duration, years (range)	3 (1–8)	4 (1–9)	*P*=0.991 (Mann–Whitney *U*)
FVC (% predicted)	85 (52–95)	81 (53–98)	*P*=0.981 (Mann–Whitney *U*)
DlCO (% predicted)	51 (32–73)	50 (32–66)	*P*=0.814 (Mann–Whitney *U*)
ILD (by HRCT)	46.1% (18/39)	45.5% (5/11)	*P*=1.00 (Fischer exact test)

ACA, anti-centromere antibodies; ARA, anti-RNA polymerase III antibodies; ATA, anti-topoisomerase I antibodies; DLCO, diffusing capacity for carbon monoxide; FVC, forced vital capacity; HRCT, high-resolution computed tomography; ILD, interstitial lung disease; TNC, tenascin-C.

Disease duration (interval from first non-Raynaud disease manifestation to sampling). Median values and interquartile ranges are shown. MRSS, 1–51.

**Table 4:** Clinical characteristics of Cohort 5.

	Healthy, *N*=22	SSc, *N*=38	*P* value
Age, mean±s.d.	48.5±12.3	50.3±11.4	0.58
Sex, *n* (% female)	86%	76%	0.35
Race, *n* (% white)	64%	74%	0.41
Disease duration, months, mean±s.d.	NA	55.6±11.4	NA
SSc subtype (% diffuse)	NA	99%	NA
Tenascin-C level, ng ml^−1^, mean±s.d.	410.1±150.4	731.2±371.5	<.0001
MRSS	NA	22 (3–40)	0.14
FVC (% predicted)	NA	81.1±20.0	0.32
DLCO (% predicted)	NA	65.5+21.0	0.05

DLCO, diffusing capacity for carbon monoxide; FVC, forced vital capacity; NA, not applicable.

MRSS, 1–51.
