# Supplementary material for: Tenascin-C drives persistence of organ fibrosis
Source: Nat Commun. 2016 Jun 3;7:11703. doi: 10.1038/ncomms11703 (PMC4895803; doi:10.1038/ncomms11703)
Supplement: Supplementary Information — Supplementary Figures 1-11 and Supplementary Tables 1-6. [file ncomms11703-s1.pdf]

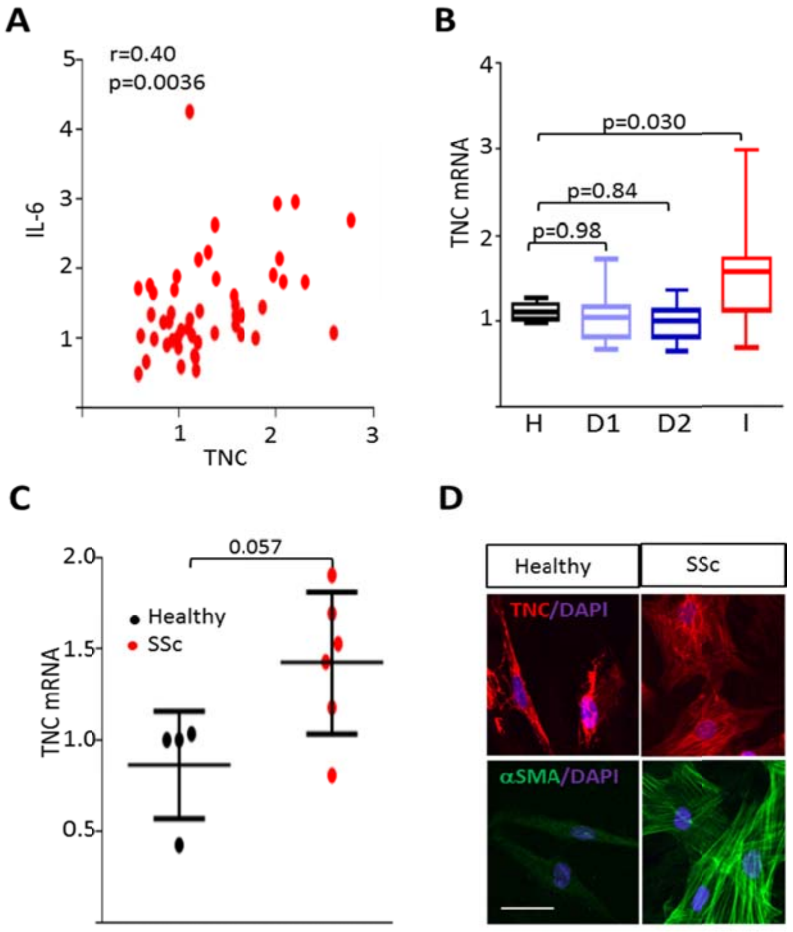


**Supplementary Fig. 1.**

**Elevated tenascin-C in SSc skin biopsies and isolated skin fibroblasts.**

**A.** Correlation of tenascin-C and IL-6 mRNA levels in an SSc biopsy-derived transcriptome dataset (GSE32413; see Fig 1 for full description). **B.** Transcriptome dataset from an independent cohort of 17 SSc and 6 healthy control skin biopsies (GSE9285). Tenascin-C mRNA levels in intrinsic subsets (H, healthy controls; D1, diffuse 1; D2, diffuse 2; I, inflammatory) shown as box plots spanning values from 25-75 percentile. Horizontal lines represent median, maximum and minimum values. One-way ANOVA followed by Sidak's multiple comparison test. **C**. RNA from healthy control (n=4) or SSc (n=6) skin fibroblasts was

examined by qPCR. Results are means ±S.D. from triplicate determination. Mann-Whitney *U* test**. D.** Immunofluorescence of explanted SSc (n =3) and healthy control (n = 2) skin fibroblasts using antibodies to tenascin-C and αSMA; nuclei identified using DAPI. Representative confocal images; bar =25 μm.

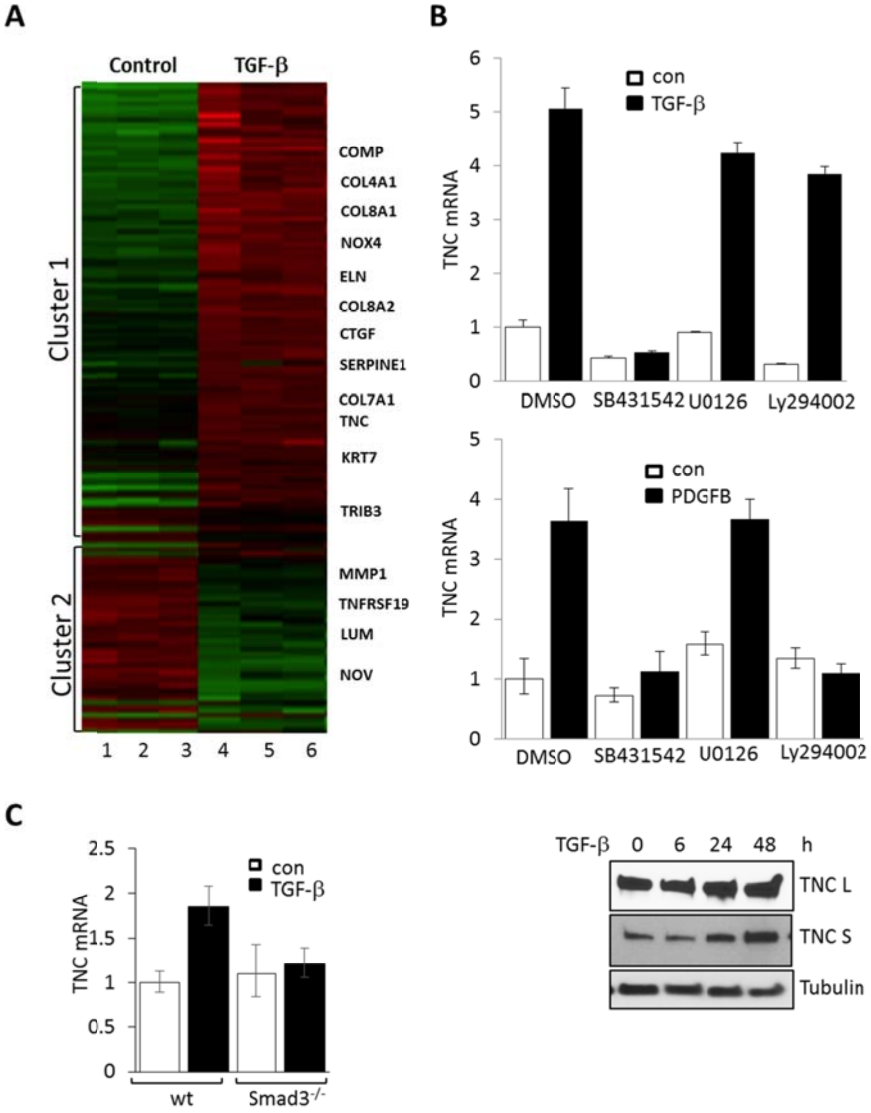


**Supplementary Fig. 2.**

**Regulation of tenascin-C expression in skin fibroblasts**

**A.** RNA isolated from healthy adult skin fibroblasts incubated with or without TGF-ß1 for 24 h was hybridized to Illumina human HT-12 Microarray Chips. Heatmap showing -fold change in gene expression compared with the average in control samples. Red, up-regulated; green, down-regulated. Lanes 1-3, untreated control fibroblasts; lanes 4-6, TGF-ß-treated fibroblasts

(triplicates). FDR 0.05, >1.5-fold change. Clusters indicate genes significantly up- or down-regulated. **B**. Foreskin fibroblasts were incubated with TGF-ß (10 ng/ml) (upper panel) or PDGFB (25 ng/ml) (lower panel) alone, or in the presence or absence of SB431532 or U0126 or Ly294002. Results of real-time qPCR represent means ± S.D. of triplicate determinations from a representative experiment. Closed bars, treated fibroblasts; open bars, untreated fibroblasts. **C**. Confluent cultures of wt and SMAD3$^{-/-}$ MEFs were incubated with TGF-ß (10 ng/ml) for 24h. Results of real-time qPCR represent means ± S.D. of triplicate determinations from a representative experiment. Closed bars, treated fibroblasts; open bars, untreated fibroblasts. **D.** Confluent adult skin fibroblasts were incubated with TGF-ß (10 ng/ml). Whole cell lysates were examined by Western analysis. S, secreted; L, lysates.

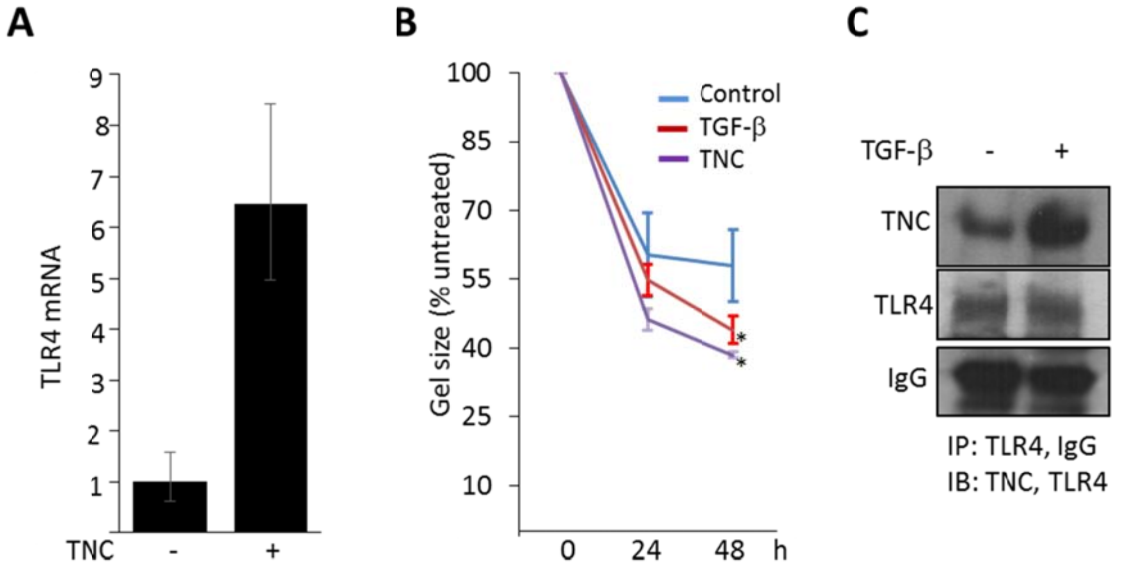


**Supplementary Fig. 3.**

**Tenascin-C enhances fibroblast contractility, induces TLR4 expression and interacts directly with TLR4.**

Confluent foreskin fibroblasts were incubated with tenascin-C (2 μg/ml) and/or TGF-ß1. **A.** Results of qPCR shown as means ± SD from triplicate determinations. **B.** Fibroblasts seeded in Type I collagen gels were incubated in media with TGF-ß in the presence or absence of tenascin-C (2 μg/ml). At 24 and 48 h, gel diameters were determined. Results, expressed as percentage of gel area compared to controls (time 0) are the mean ± S.D. of triplicate determinations from three separate wells. Mann-Whitney *U* test**.** **C.** Whole-cell lysates were immunoprecipitated with antibodies to TLR4 or IgG, followed by immunobloting with antibodies to tenascin-C or TLR4. Representative immunoblot.

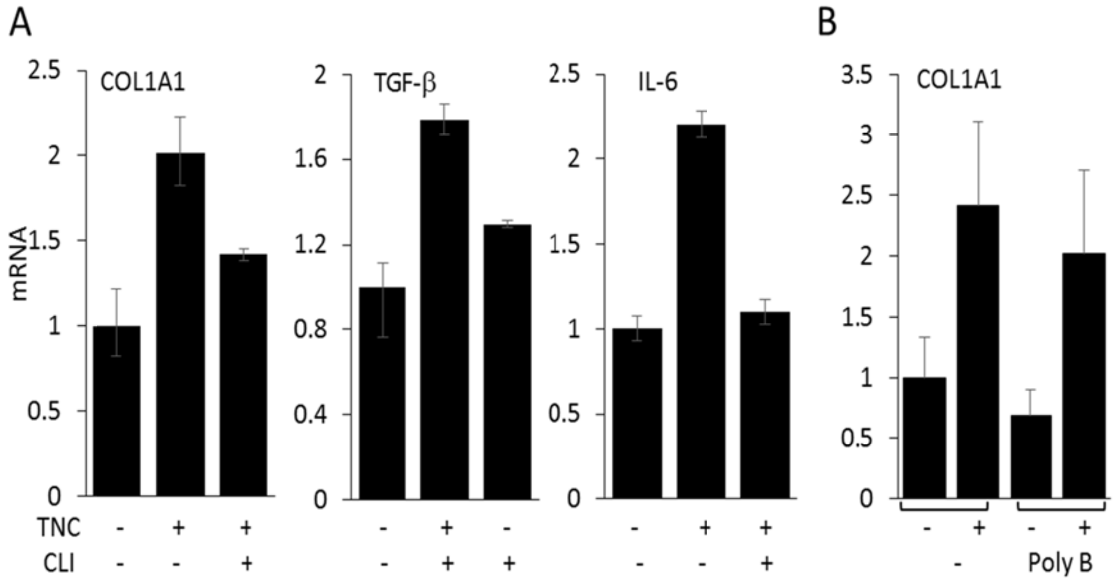


**Supplementary Fig. 4.**

**Tenascin-C-induced fibrotic responses are TLR4-dependent**

Foreskin fibroblasts were incubated in media with tenascin-C (2 μg/ml) in the absence or presence of CLI-095 for 72 h (A), or polymyxin B for 24 h (B). qPCR results shown as means ± S.D. from triplicate determinations from representative experiments.

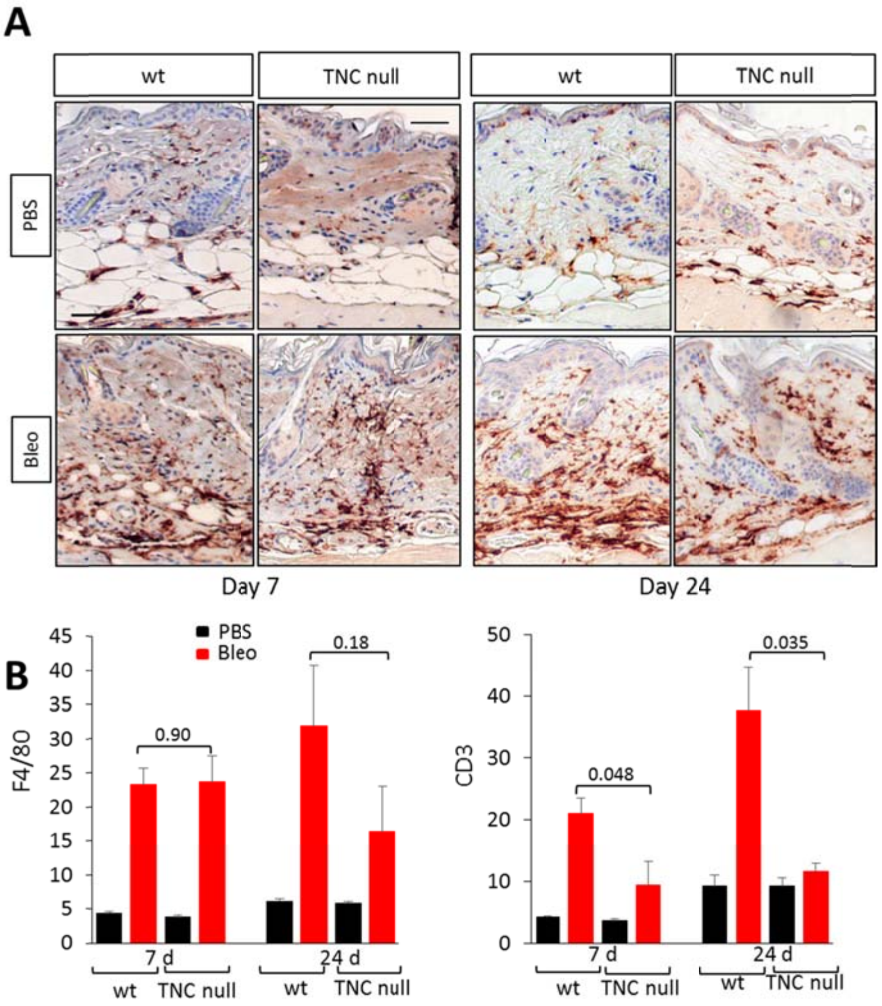


**Supplementary Fig. 5.**

**Bleomcyin-induced macrophage and T cell accumulation in skin in wildtype and TNC$^{-/-}$ mice.**

Wildtype mice and TNC$^{-/-}$ mice received belomycin or PBS via s.c. injections. Skin was harvested at day 7 and day 24. **A.** Immunohistochemistry using antibodies to F4/80. Representative photomicrographs. Bars, 50 µm. **B.** Positive immunostaining quantified using Image J. Results (percentage of area stained positive for F4/80 or CD3 from three randomly selected areas) are means ± S.D. of three mice/group. Student's t-test.

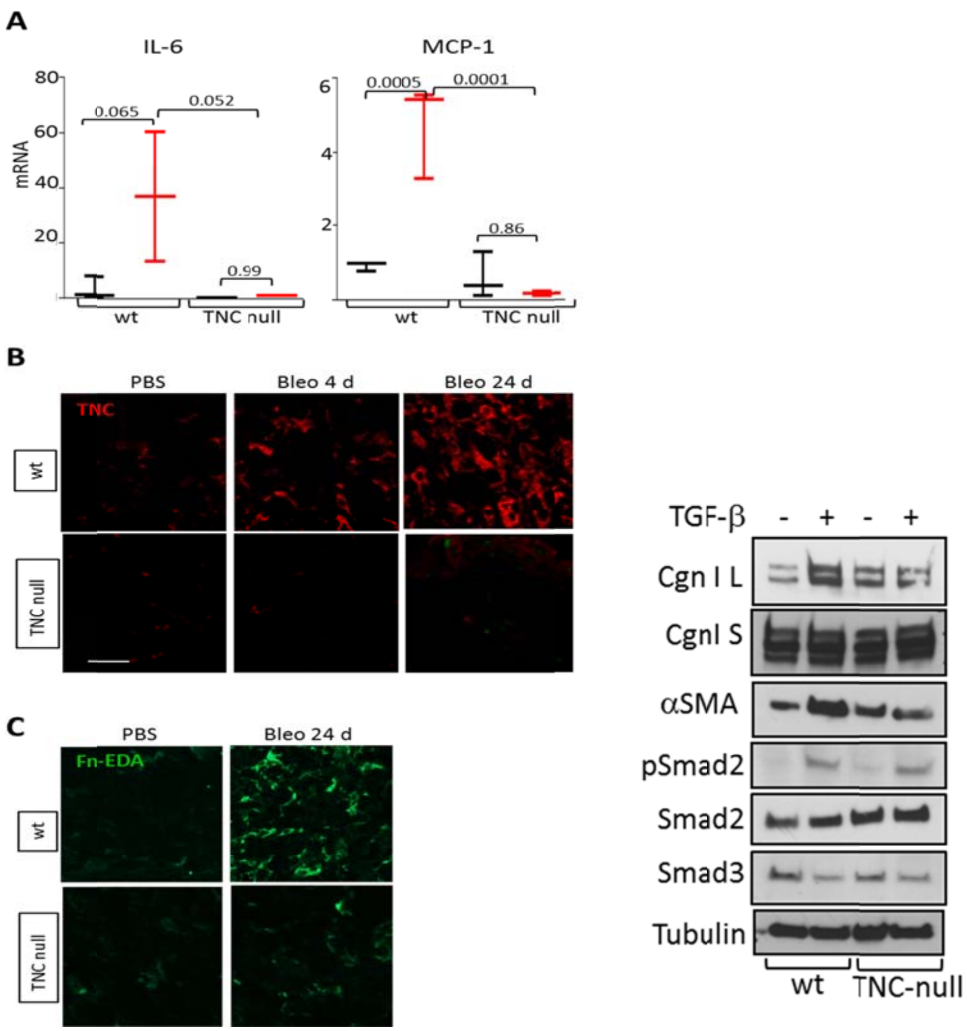


**Supplementary Fig. S6**

**Attenuated cutaneous inflammation and Fn-EDA and tenascin-C deposition in *TNC$^{-/-}$* mice.** Wildtype mice and TNC$^{-/-}$ mice were administered bleomycin or PBS via s.c. injections. Skin was harvested at day 14 (A) or 24 (B). **A.** Results of qPCR, normalized with GAPDH, are means ± S.D. of triplicate determinations from three mice/group. **B, C.** Immunofluorescence microscopy using antibodies to tenascin-C or Fn-EDA. Representative images; bars = 25 µm.

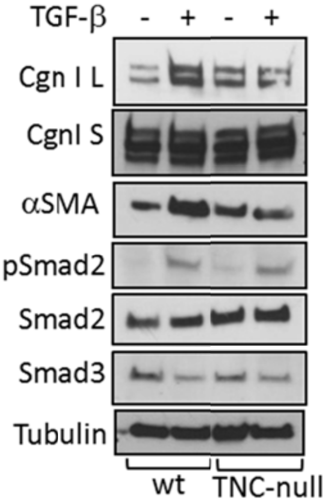


**Supplementary Fig. 7**.

**Impaired TGF-ß responses in TNC$^{-/-}$ skin fibroblasts.**

Confluent skin fibroblasts isolated from wildtype mice and *TNC*-/- mice were incubated in parallel with TGF-β for 24 h. Whole cell lysates were examined Western blot analysis. Representative immunoblot. S, secreted; L, lysates.

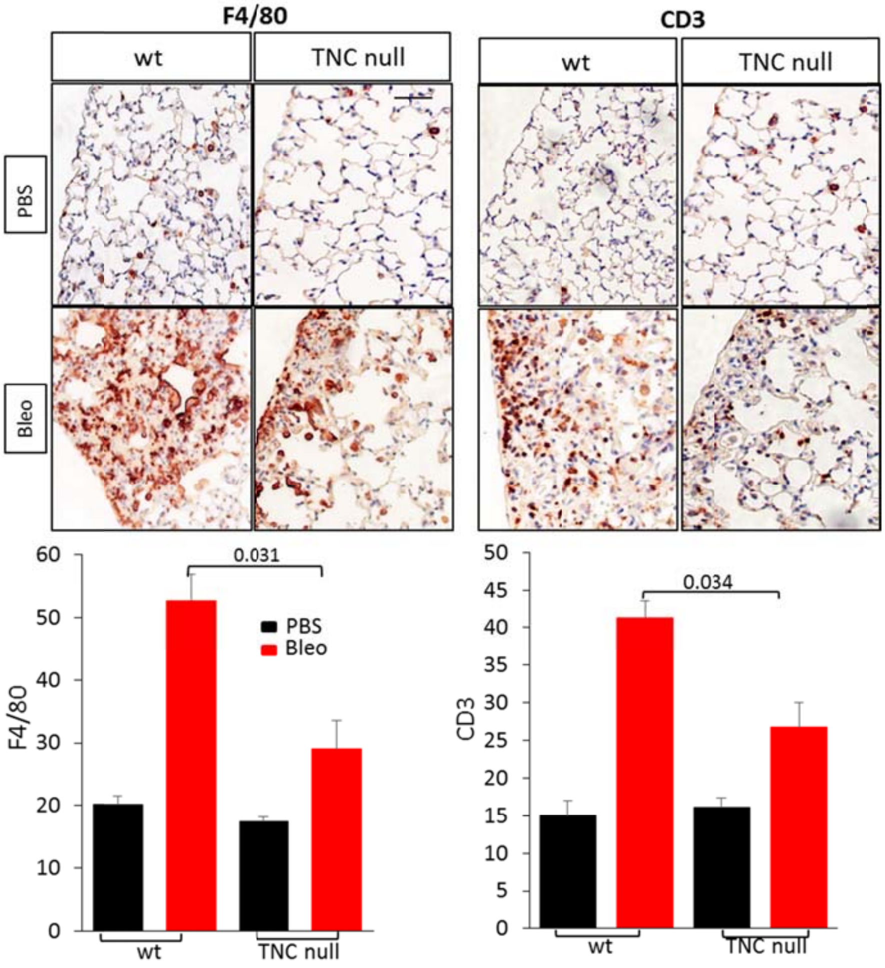


**Supplementary Fig. 8.**

**Bleomycin-induced macrophage accumulation in the lungs in wildtype and TNC$^{-/-}$ mice.**

Wildtype mice and TNC$^{-/-}$-null mice were administered bleomcyin via s.c. injections, and lungs were harvested at day 24. Upper panels, immunohistochemistry using antibodies to F4/80 or CD3. Representative photomicrographs. Bars, 50 µm. Lower panels, positive immunostaining was quantified using Image J; results (percentage of area stained positive for F4/80 or CD3) are means ± S.D. of percentage of area stained positive for F4/80 or CD3 from three randomly chosen fields from a single mouse representative of 3 mice. Student's t-test.

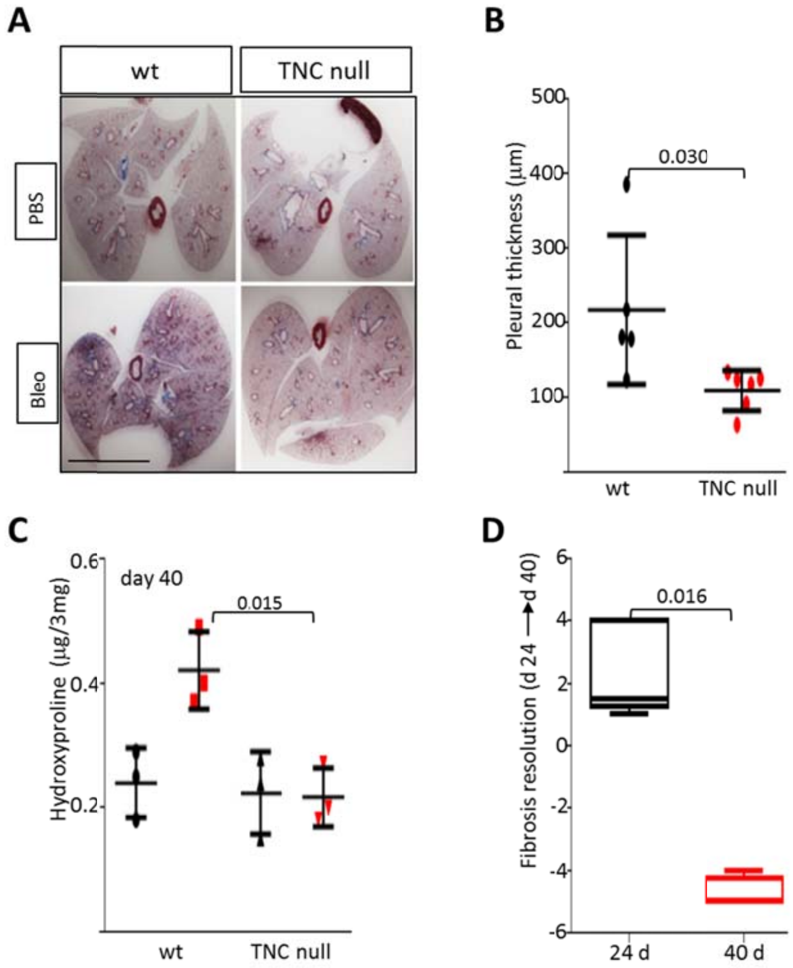


**Supplementary Fig. 9.**

**Attenuated lung fibrosis and accelerated resolution in *TNC*$^{-/-}$ mice.**

Wildtype mice and TNC$^{-/-}$ mice were administered bleomcyin via s.c. injections, and lungs were harvested at day 24 (A,B) or day 40 (C,D). **A.** Trichrome stain. Representative images. Bars = 0.2 mm. **B.** Pleural thickness. Mann-Whitney *U* test**. C**. Lung collagen content at 40 d determined by hydroxyproline assays. Mann-Whitney *U* test**.** Results are the means ± SD of triplicate determinations from 3 mice/group. **D**. Fibrosis resolution from day 24 to day 40. Mann-Whitney *U* test**.**

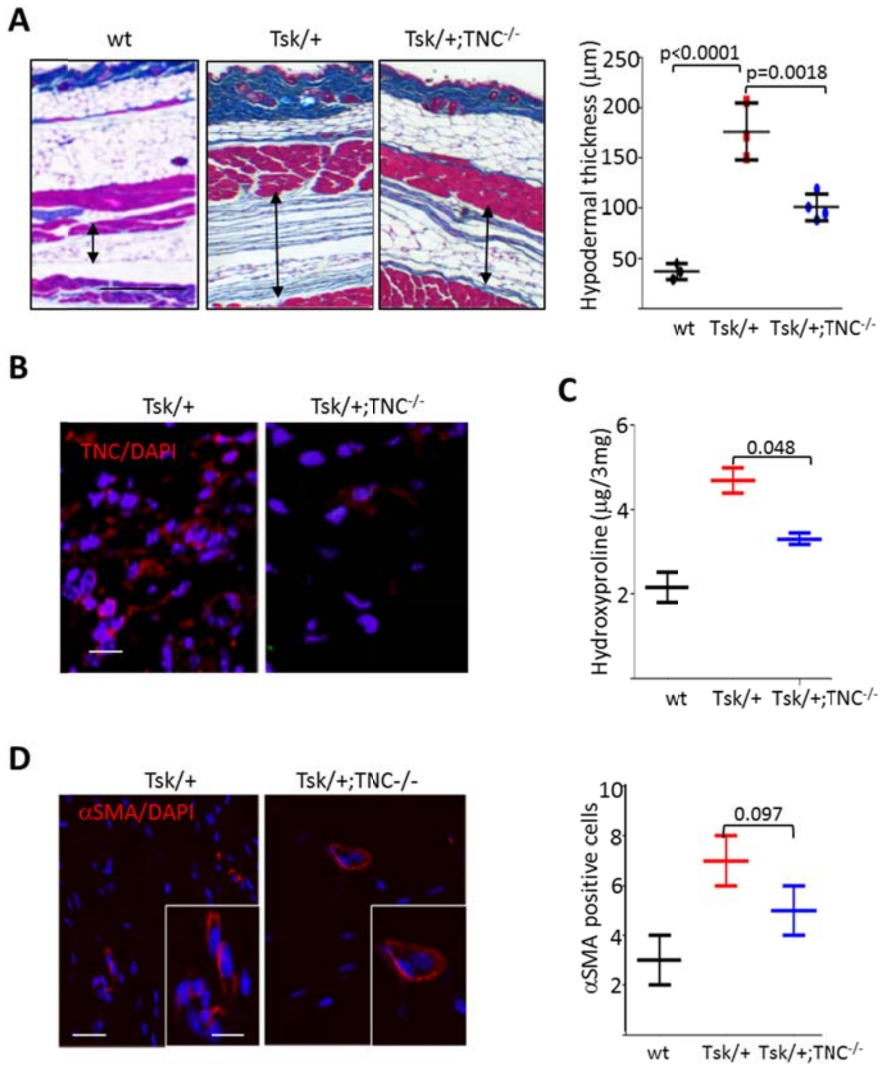


**Supplementary Fig. 10.**

**Attenuated hypodermal fibrosis in TSK/+ mice lacking tenascin-C**

Mice were sacrificed at 12 weeks of age, and skin including hypodermis was harvested. **A.** Trichrome stain. Arrows indicate hypodermis. Bar, 100 µm. Representative images from 2-3 mice/group. Right panel, hypodermal thickness. Results are means ± SD from 5 hpf/mouse. One-way ANOVA followed by Sidak's multiple comparison test. **B, D**. Immunofluorescence microscopy using antibodies to tenascin-C or αSMA; DAPI used to identify nuclei. Bar, 25 µm (B) or 50 µm (D). Student's t-test. **C.** Collagen content determined by hydroxyproline assays.

Results are the means ± S.D. from triplicate determinations from 2 mice/group. Right panel, quantitation of αSMA-immunopositive interstitial cells within the hypodermis. Results are the means ± S.D. of three determinations from 3 randomly chosen hpf. Student's t-test.

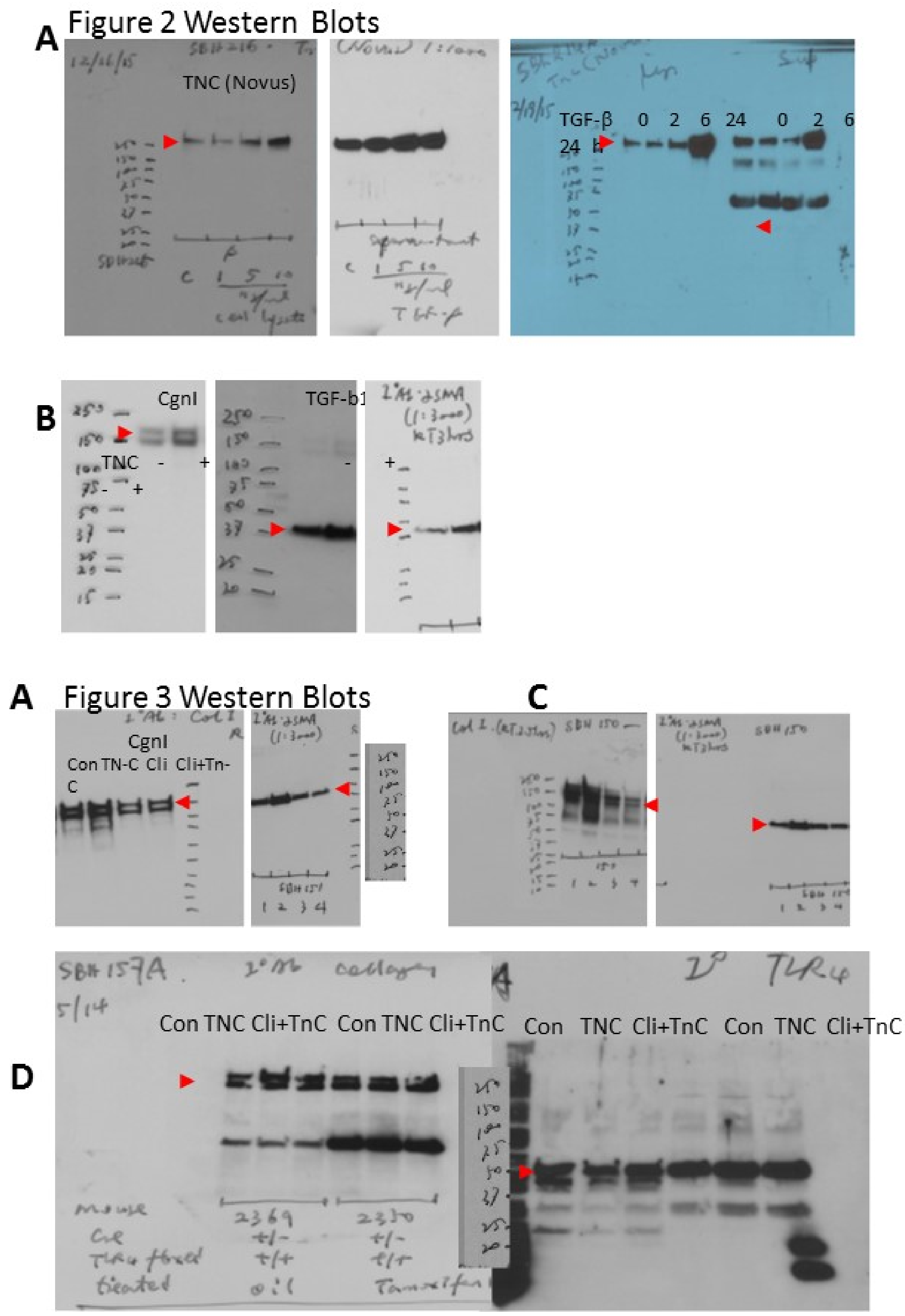


**Supplementary Fig. 11.** The full Western blots of Figs. 2 and 3.

## Supplementary Table 1

## Expression of endogenous TLR4 ligands in skin biopsies

| Endogenous TLR ligand | Expression in SSc skin | Expression in healthy skin |
|---|---|---|
| Fibronectin-EDA | ++++ | - |
| Tenascin-C | ++++ | - |
| HA (low molecular weight) | ++ | - |
| Biglycan | variable | - |
| GP96 | - | - |
| HMGB1 | - | - |

Forearm skin biopsies from SSc patients (n=3) and healthy controls (n=3) were examined by immunofluorescence and/or immunohistochemistry. Staining intensity in the dermis for each antibody was scored by two blinded observers as follows: -, undetectable in the interstitial space; +, weak expression; ++, strong expression; +++, very strong expression.

## Supplementary Table 2 (Cohort 4)

| Subject code | Age | Gender | Subtype | MRSS | scl-70 | RNA Th/To | RNA U11. |
|---|---|---|---|---|---|---|---|
| | | | | | | | |
| 874 | 46 | F | lcSSc | 13 | 1 | NA | NA |
| 975 | 27 | F | dcSSc | 14 | 1 | NA | NA |
| 1007 | 25 | F | lcSSc | 10 | 1 | NA | NA |
| 1194 | 54 | F | lcSSc | 2 | 0 | 1 | NA |
| 1219 | 36 | F | lcSSc | 3 | 0 | 1 | NA |
| 1556 | 27 | F | lcSSc | 0 | 1 | 0 | NA |
| 1602 | 32 | F | dcSSc | 13 | 1 | 0 | NA |
| 1661 | 31 | F | lcSSc | 4 | 1 | 0 | NA |
| 1857 | 20 | F | dcSSc | 12 | 1 | 0 | NA |
| 1939 | 18 | F | lcSSc | 3 | 1 | NA | NA |
| 2039 | 42 | F | lcSSc | 17 | 1 | NA | NA |
| 2136 | 46 | F | lcSSc | 0 | 0 | 1 | NA |
| 2221 | 27 | F | dcSSc | 14 | 1 | 0 | NA |
| 2244 | 28 | F | lcSSc | 10 | 1 | NA | NA |
| 2259 | 20 | F | lcSSc | 3 | 1 | NA | NA |
| 2302 | 42 | F | dcSSc | 2 | 0 | 1 | NA |
| 2448 | 41 | F | lcSSc | 6 | 0 | 1 | NA |
| 2457 | 36 | F | lcSSc | 13 | 1 | NA | NA |
| 2620 | 37 | F | lcSSc | 7 | 0 | NA | 1 |
| 2635 | 42 | F | lcSSc | 32 | 1 | NA | NA |
| 2725 | 43 | F | lcSSc | 17 | 1 | NA | NA |
| 2748 | 23 | F | lcSSc | 10 | 1 | NA | NA |
| 2749 | 28 | F | dcSSc | 2 | 0 | 1 | NA |
| 2776 | 33 | F | lcSSc | 18 | 1 | NA | NA |
| 2781 | 43 | F | lcSSc | 17 | 1 | NA | NA |
| 2819 | 44 | F | dcSSc | 27 | 1 | NA | NA |
| 2821 | 46 | F | dcSSc | 4 | 0 | 1 | NA |
| 2834 | 36 | F | lcSSc | 8 | 1 | NA | NA |
| 2919 | 49 | F | lcSSc | 21 | 1 | NA | NA |
| 2962 | 22 | F | dcSSc | 6 | 1 | NA | NA |
| 3049 | 45 | F | dcSSc | 5 | 0 | NA | 1 |
| 3098 | 36 | F | dcSSc | 2 | 1 | NA | NA |
| 3137 | 42 | F | lcSSc | 27 | 1 | NA | NA |
| 3211 | 54 | F | lcSSc | 27 | 1 | NA | NA |
| 3224 | 30 | F | dcSSc | 4 | 0 | 1 | NA |
| 3339 | 41 | F | lcSSc | 7 | 1 | NA | NA |
| 3340 | 30 | F | lcSSc | 2 | 1 | NA | NA |

| | | | | | | | |
|---|---|---|---|---|---|---|---|
| 3354 | 17 | F | dcSSc | 20 | 1 | NA | NA |
| 3428 | 35 | F | lcSSc | 6 | 1 | NA | NA |
| 3433 | 45 | F | lcSSc | 4 | 0 | 1 | NA |
| 3486 | 30 | F | dcSSc | 43 | 1 | NA | NA |
| 3487 | 35 | F | lcSSc | 6 | 1 | NA | NA |
| 3633 | 37 | F | lcSSc | 6 | 1 | NA | NA |
| 3645 | 29 | F | lcSSc | 10 | 1 | NA | NA |
| 3663 | 28 | F | lcSSc | 6 | 1 | NA | NA |
| 3665 | 50 | F | lcSSc | 4 | 1 | NA | NA |
| 3835 | 26 | F | dcSSc | 1 | 0 | 1 | NA |
| 3843 | 25 | F | dcSSc | 14 | 1 | NA | NA |
| 3880 | 46 | F | lcSSc | 1 | 1 | NA | NA |
| 3919 | 36 | F | lcSSc | 0 | 0 | 1 | NA |
| 3920 | 50 | F | lcSSc | 4 | 1 | 0 | NA |
| 3942 | 56 | F | dcSSc | 9 | 1 | NA | NA |
| 3954 | 51 | F | lcSSc | 3 | 0 | 1 | NA |
| 3981 | 48 | F | lcSSc | 8 | 0 | 1 | NA |
| 4054 | 48 | F | dcSSc | 14 | 1 | NA | NA |
| 4175 | 35 | F | dcSSc | 10 | 1 | NA | NA |
| 4189 | 31 | F | lcSSc | 1 | 0 | 0 | 1 |
| 4198 | 48 | F | dcSSc | 20 | 1 | NA | NA |
| 4224 | 44 | F | dcSSc | 39 | 0 | 0 | 1 |
| 4226 | 37 | F | dcSSc | 24 | 0 | NA | 1 |
| 4378 | 51 | F | lcSSc | 7 | 1 | NA | NA |

Subjects providing serum for tenascin-C determination (validation Cohort) (all female). dcSSc, diffuse cutaneous SSc; lcSSc, limited cutaneous SSc; M, male, F, female. MRSS, modified Rodnan skin score (1-51). 0 represents antibody not present, 1 represents antibody present, NA, not available. Controls were healthy subjects (90% female; median age, 38 years; range, 18 to 54 years).

## Supplementary Table 3 (Cohort 5)

| Subject Code | Age | Gender | Subtype | MRSS | Positive ANA | scl-70 | ACA | RNA pol III |
|---|---|---|---|---|---|---|---|---|
| GC-01-SN | NA | M | dcSSc | 19 | 1 | 0 | NA | NA |
| GC-02-SN | NA | F | dcSSc | 31 | 1 | 0 | NA | NA |
| GC-04-SN | NA | F | dcSSc | 26 | 1 | 0 | NA | NA |
| GC-05-SN | NA | M | dcSSc | 39 | 1 | 0 | NA | NA |
| GC-08-SN | NA | F | dcSSc | 23 | 1 | 0 | NA | NA |
| GC-09-SN | NA | M | dcSSc | 39 | 1 | 1 | NA | NA |
| SR1002_ | 34 | F | dcSSc | 32 | 1 | 1 | 0 | NA |
| SR1010 | 60 | F | dcSSc | 30 | 1 | 0 | 0 | 1 |
| SR1024 | 56 | F | dcSSc | 31 | 1 | 0 | 0 | 0 |
| SR1038 | 63 | F | dcSSc | 32 | 1 | 0 | 0 | |
| SR1042 | 48 | F | dcSSc | 8 | 1 | 1 | 0 | 0 |
| SR1067 | 58 | F | dcSSc | 12 | 1 | 0 | 0 | 0 |
| SR1080 | 56 | F | dcSSc | 22 | 1 | 0 | 0 | 1 |
| SR1094 | 54 | F | dcSSc | 15 | 1 | 0 | 1 | |
| SR1107 | 50 | M | dcSSc | 4 | 1 | 0 | 0 | 0 |
| SR1121 | 47 | F | dcSSc | 26 | 1 | 0 | 0 | 1 |
| SR1157 | 52 | F | dcSSc | 18 | 1 | 0 | 0 | 0 |
| SR1209 | 54 | F | dcSSc | 32 | 1 | 0 | 0 | 1 |
| SR1213 | 30 | F | lcSSc | 10 | 1 | 1 | 0 | 0 |
| SR1241 | 55 | M | dcSSc | 19 | 1 | 0 | 0 | 1 |
| SR1366 | 55 | F | dcSSc | 23 | 1 | 0 | 0 | 2 |
| SR1381 | 69 | F | dcSSc | 12 | 1 | 1 | 0 | 0 |
| SR1393 | 41 | F | dcSSc | 14 | 1 | 0 | 0 | 0 |
| SR1420 | 49 | F | dcSSc | 28 | 1 | 0 | 0 | 1 |
| SR1430 | 44 | F | dcSSc | 4 | 0 | 0 | 0 | 0 |
| S-Y0-164 | NA | F | dcSSc | 3 | 1 | 1 | NA | NA |
| S-Y0-187 | NA | M | dcSSc | 15 | 1 | 0 | NA | NA |
| S-Y0-208 | NA | M | dcSSc | 20 | 1 | 1 | NA | NA |
| S-Y0-220 | NA | F | dcSSc | | 1 | 1 | NA | NA |
| S-Y0-235 | NA | F | dcSSc | 16 | 1 | 1 | NA | NA |

| | | | | | | | | |
|---|---|---|---|---|---|---|---|---|
| S-Y0-257 | NA | F | dcSSc | 22 | 1 | NA | NA | NA |
| S-Y0-261 | NA | F | dcSSc | 33 | 1 | 0 | NA | NA |
| S-Y1-088 | NA | F | dcSSc | 37 | 1 | 0 | NA | NA |
| S-Y1-176 | NA | F | dcSSc | 11 | 1 | 1 | NA | NA |
| S-Y1-179 | NA | M | dcSSc | 28 | 1 | 0 | NA | NA |
| S-Y1-183 | NA | F | dcSSc | 40 | 1 | NA | NA | NA |
| S-Y1-25 | NA | M | dcSSc | 26 | 1 | NA | NA | NA |
| S-Y1-79 | | F | dcSSc | 32 | | NA | NA | NA |

Subjects providing serum for tenascin-C determination (validation Cohort). dcSSc, diffuse cutaneous SSc; lcSSc, limited cutaneous SSc; M, male, F, female. MRSS, modified Rodnan skin score (1-51). 0 represents antibody not present, 1 represents antibody present, NA, not available. Controls were healthy subjects (90% female; median age, 38 years; range, 18 to 54 years).

## Supplementary Table 4A.

## Top 30 genes up-regulated by TGF-ß in normal fibroblasts

| Gene name | Abbreviation | Fold-induction | p value |
|---|---|---|---|
| ASNS | asparagine synthetase (glutamine-hydrolyzing) | 2.99 | 1.07E-13 |
| PSAT1 | phosphoserine aminotransferase 1 | 2.69 | 1.05E-11 |
| COMP | cartilage oligomeric matrix protein | 16.3 | 2.32E-11 |
| INHBE | inhibin, beta E | 4.94 | 3.32E-11 |
| LTBP2 | latent transforming growth factor beta binding protein 2 | 3.03 | 3.86E-11 |
| CNN1 | calponin 1, basic, smooth muscle | 3.43 | 5.79E-11 |
| HSD17B6 | hydroxysteroid (17-beta) dehydrogenase 6 homolog (mouse) | 2.78 | 1.20E-10 |
| BHLHE40 | basic helix-loop-helix family, member e40 | 3.53 | 2.74E-10 |
| ACTG2 | actin, gamma 2, smooth muscle, enteric | 2.54 | 6.89E-10 |
| KCNMB1 | potassium large conductance calcium-activated channel, subfamily M, beta member 1 | 2.59 | 7.48E-10 |
| KRT7 | keratin 7 | 3.87 | 9.31E-10 |
| MGP | matrix Gla protein | 3.3 | 3.68E-09 |
| TPM1 | tropomyosin 1 (alpha) | 2.48 | 6.08E-09 |
| ITGA11 | integrin, alpha 11 | 2.96 | 1.17E-08 |
| CDH2 | cadherin 2, type 1, N-cadherin (neuronal) | 2.39 | 2.46E-08 |
| MTHFD2 | methylenetetrahydrofolate dehydrogenase (NADP+ dependent) 2, methenyltetrahydrofolate cyclohydrolase | 2.62 | 2.74E-08 |
| NOX4 | NADPH oxidase 4 | 3.9 | 2.91E-08 |
| PI16 | peptidase inhibitor 16 | 4.51 | 3.01E-08 |
| TRIB3 | tribbles homolog 3 (Drosophila) | 3.69 | 4.56E-08 |
| NUAK1 | NUAK family, SNF1-like kinase, 1 | 2.63 | 4.82E-08 |
| CTPS | CTP synthase | 2.41 | 9.39E-08 |
| ***TN-C*** | ***tenascin-C*** | ***2.59*** | ***9.70E-08*** |
| ALDH1B1 | aldehyde dehydrogenase 1 family, member B1 | 2.49 | 1.68E-07 |
| SERPINE1 | serpin peptidase inhibitor, clade E (nexin, plasminogen activator inhibitor type 1), member 1 | 2.52 | 2.23E-07 |
| SLC7A5 | solute carrier family 7 (cationic amino acid transporter, y+ system), member 5 | 2.82 | 3.17E-07 |
| ELN | elastin | 3.98 | 1.03E-06 |
| WFDC1 | WAP four-disulfide core domain 1 | 2.5 | 1.29E-05 |
| SLC2A5 | solute carrier family 2 (facilitated glucose/fructose transporter), member 5 | 2.65 | 0.000026 |
| PMEPA1 | prostate transmembrane protein, androgen induced 1 | 2.54 | 3.66E-05 |
| KANK4 | KN motif and ankyrin repeat domains 4 COMP | 3.03 | 0.000122 |
| ACTC1 | actin, alpha, cardiac muscle 1 | 2.7 | 0.000277 |

**Supplementary Table 4B.**

## Top 30 genes down-regulated by TGF-ß in normal fibroblasts

| Gene name | Abbreviation | Fold-induction | p value |
|---|---|---|---|
| APCDD1 | adenomatosis polyposis coli down-regulated 1 | -3.43 | 1.80E-11 |
| SLC7A14 | solute carrier family 7 (cationic amino acid transporter, y+ system), member 14 | -2.58 | 3.13E-11 |
| NOV | nephroblastoma overexpressed gene | -4.36 | 5.07E-11 |
| ADH1A | alcohol dehydrogenase 1A (class I), alpha polypeptide | -2.04 | 2.04E-10 |
| RAB38 | RAB38, member RAS oncogene family | -2.16 | 2.88E-10 |
| MMP1 | matrix metallopeptidase 1 (interstitial collagenase) | -3.06 | 5.96E-10 |
| TYMS | thymidylate synthetase | -2.12 | 1.13E-09 |
| NPTX1 | neuronal pentraxin I | -5.97 | 2.16E-09 |
| GPM6B | glycoprotein M6B | -2.28 | 2.20E-09 |
| PDGFRL | platelet-derived growth factor receptor-like | -2.29 | 2.55E-09 |
| PCOLCE2 | procollagen C-endopeptidase enhancer 2 | -2.61 | 3.28E-09 |
| CLEC3B | C-type lectin domain family 3, member B | -3.51 | 5.84E-09 |
| HSD17B2 | hydroxysteroid (17-beta) dehydrogenase 2 | -2.4 | 1.17E-08 |
| TNFRSF19 | tumor necrosis factor receptor superfamily, member 19 | -2.02 | 2.18E-08 |
| COLEC12 | collectin sub-family member 12 | -2.27 | 2.56E-08 |
| ANGPTL4 | angiopoietin-like 4 | -2.03 | 4.72E-08 |
| PDGFD | platelet derived growth factor D | -2.46 | 6.75E-08 |
| OLFML2A | olfactomedin-like 2A | -2 | 3.17E-07 |
| OLFML1 | olfactomedin-like 1 | -2.09 | 6.53E-07 |
| OAF | OAF homolog (Drosophila) | -2.17 | 7.93E-07 |
| BMP4 | bone morphogenetic protein 4 | -2.12 | 9.29E-07 |
| SNHG7 | small nucleolar RNA host gene 7 (non-protein coding) | -2.01 | 1.23E-06 |
| CLDN11 | claudin 11 | -2.79 | 1.25E-06 |
| NFIA | nuclear factor I/A | -2.01 | 2.41E-06 |
| RSPO3 | R-spondin 3 homolog (Xenopus laevis) | -2 | 2.84E-06 |
| TGFBR3 | transforming growth factor, beta receptor III | -2.18 | 3.99E-06 |
| CCRL1 | chemokine (C-C motif) receptor-like 1 | -2.32 | 4.46E-06 |
| NTN4 | netrin 4 | -2.06 | 3.43E-05 |
| APOD | apolipoprotein D | -2.26 | 3.98E-05 |
| RGS2 | regulator of G-protein signaling 2, 24kDa | -2.19 | 1.07E-04 |
| LUM | Lumican | -2.3 | 3.51E-03 |

Dermal fibroblasts were incubated with TGF-ß for 24 h, and RNA was isolated and hybridized to Illumina human HT-12 v4 Expression Microarray Chips. The -fold change of gene expression compared with the average in control samples are shown.

## Supplementary Table 5

## Modulation of lung mechanics by bleomcyin

Lung mechanics were evaluated on day 24 in wildtype and $TNC^{-/-}$ mice injected with s.c. PBS or bleomycin. Results represent means ± s.d. from at least eight mice/group  E, dynamic elastance; C, dynamic compliance; R, dynamic resistance; Rn, Newtonian resistance; G, tissue damping; H, tissue elastance; eta, tissue hysteresivity; Area, area enclosed by pressure volume loop. *p<0.05.

| | Wildtype mice PBS | Wtildtype mice Bleo | $TNC^{-/-}$ mice PBS | $TNC^{-/-}$ mice Bleo | p |
|---|---|---|---|---|---|
| C cmH2O/mL | 0.024±0.001 | 0.016±0.004 | 0.024±0.003 | 0.021±0.002 | 0.0059* |
| E cmH2O.s/mL | 41.41±1.76 | 71.35±41.98 | 41.58±6.08 | 47.19±5.77 | 0.072 |
| R cmH2O.s/mL | 0.90±0.05 | 1.25±0.67 | 0.94±0.16 | 0.98±0.12 | 0.30 |
| Rn cmH2O.s/mL | 0.34±0.14 | 0.35±0.17 | 0.34±0.15 | 0.35±0.13 | 0.99 |
| G cmH2O/mL | 7.76±0.57 | 11.94±7.21 | 8.07±1.07 | 8.45±1.03 | 0.16 |
| H cmH2O/mL | 44.46±3.15 | 74.65±42.03 | 41.79±6.46 | 46.35±5.93 | 0.028* |
| Eta | 0.17±0.017 | 0.16±0.023 | 0.190±.02 | 0.18±0.01 | 0.031* |
| Area cmH2O.mL | 2.55±1.39 | 4.19±1.56 | 2.71±1.03 | 2.62±0.94 | 0.023* |

## Supplementary Table 6

### Primer sequences used for qPCR analysis

| | | |
|---|---|---|
| **Human**: | | |
| | | |
| hCOL1A1 | Forward | 5'-TGGTGTGCAAGGTCCC-3' |
| | Reverse | 5'-CATTCCCTGAAGGCCAG-3' |
| | | |
| hCOL1A2 | Forward | 5'-CGGACGACCTGGTGAGAGA-3' |
| | Reverse | 5'-CATTGTGTCCCCTAATGCCTT-3' |
| | | |
| h-SMA | Forward | 5'-CAGGGCTGTTTTCCCATCCAT-3' |
| | Reverse | 5'-GCCATGTTCTATCGGGTACTTC-3' |
| | | |
| hIL6 | Forward | 5'-AAATTCGGTACATCCTCGACGG-3' |
| | Reverse | 5'-GGAAGGTTCAGGTTGTTTTCTGC-3' |
| | | |
| hTNC1 | Forward | 5’-ACCGCTACCGCCTCAATTAC-3’ |
| | Reverse | 5’-GGTTCCGTCCACAGTTACCA-3’ |
| | | |
| hTNC1 | Forward | 5’-ACCGCTACCGCCTCAATTAC-3’ |
| | Reverse | 5’-GGTTCCGTCCACAGTTACCA-3’ |
| | | |
| hTNC2 | Forward | 5’-ACTGTGGACGGAACCAAGAC-3’ |
| | Reverse | 5’-TGTGGTGAATGACCCTGAGA3’-3' |
| | | |
| hGAPDH | Forward | 5'-CATGAGAAGTATGACAACAGCCT-3' |
| | Reverse | 5'-AGTCCTTCCACGATACCAAAGT-3' |
| | | |
| | | |
| **Mouse:** | | |
| | | |
| mCOL1A1 | Forward | 5'-AGCCGCAAAGAGTCTACATG-3' |
| | Reverse | 5'-CTTAGGCCATTGTGTATGCAG-3' |
| | | |
| mCOL1A2 | Forward | 5'-CCGTGCTTCTCAGAACATCA-3' |
| | Reverse | 5'-CTTGCCCCATTCATTTGTCT-3' |
| | | |

| | | |
|---|---|---|
| mαSMA | Forward | 5'-ATGCAGAAGGAGATCACAGC-3' |
| | Reverse | 5'-GTATTCCTGTTTGCTGATCCAC-3' |
| | | |
| mFn-EDA | Forward | 5'-AGTCAGTGTGGTTGCCTTG-3' |
| | Reverse | 5'-CTGAACACTGGGTGCTATCC-3' |
| | | |
| mTGF-β1 | Forward | 5'-TACAGCAAGGTCCTTGCCCT-3' |
| | Reverse | 5'-GCAGCACGGTGACGCC-3' |
| | | |
| mIL-6 | Forward | 5'-GTACTCCAGAAGACCAGAGG-3' |
| | Reverse | 5'-TGCTGGTGACAACCACGGCC-3' |
| | | |
| mGAPDH | Forward | 5'-ATCTTCTTGTGCAGTGCCAGC-3' |
| | Reverse | 5'-GTTGATGGCAACAATCTCCAC-3' |
